# A novel interaction perturbation analysis reveals a comprehensive regulatory principle underlying various biochemical oscillators

**DOI:** 10.1186/s12918-017-0472-7

**Published:** 2017-10-10

**Authors:** Jun Hyuk Kang, Kwang-Hyun Cho

**Affiliations:** 1 Laboratory for Systems Biology and Bio-inspired Engineering, Department of Bio and Brain Engineering, Korea Advanced Institute of Science and Technology (KAIST), Daejeon, 34141 Republic of Korea; 2 Graduate School of Medical Science and Engineering, Korea Advanced Institute of Science and Technology (KAIST), Daejeon, 34141 Republic of Korea

**Keywords:** Biochemical oscillators, Network structure, Regulation of frequency and amplitude, Perturbation analysis, Systems biology

## Abstract

**Background:**

Biochemical oscillations play an important role in maintaining physiological and cellular homeostasis in biological systems. The frequency and amplitude of oscillations are regulated to properly adapt to environments by numerous interactions within biomolecular networks. Despite the advances in our understanding of biochemical oscillators, the relationship between the network structure of an oscillator and its regulatory function still remains unclear. To investigate such a relationship in a systematic way, we have developed a novel analysis method called interaction perturbation analysis that enables direct modulation of the strength of every interaction and evaluates its consequence on the regulatory function. We have applied this new method to the analysis of three representative types of oscillators.

**Results:**

The results of interaction perturbation analysis showed different regulatory features according to the network structure of the oscillator: (1) both frequency and amplitude were seldom modulated in simple negative feedback oscillators; (2) frequency could be tuned in amplified negative feedback oscillators; (3) amplitude could be modulated in the incoherently amplified negative feedback oscillators. A further analysis of naturally-occurring biochemical oscillator models supported such different regulatory features according to their network structures.

**Conclusions:**

Our results provide a clear evidence that different network structures have different regulatory features in modulating the oscillation frequency and amplitude. Our findings may help to elucidate the fundamental regulatory roles of network structures in biochemical oscillations.

**Electronic supplementary material:**

The online version of this article (10.1186/s12918-017-0472-7) contains supplementary material, which is available to authorized users.

## Background

Oscillations are commonly observed phenomena in biological systems and perform crucial functions in regulating physiological or cellular processes [1]. The beating of the heart, the breathing motion of the lungs, and the circadian rhythm of sleep and wakefulness can be regarded as oscillations to maintain physiological homeostasis [2]. Glucose metabolism, cyclic adenosine monophosphate (cAMP) generation, mitogen-activated protein kinase (MAPK) signaling, and cell cycle progression are well-known cellular oscillations [3].

Oscillators appear to have different requirements for regulating the frequency and amplitude depending on their biological functions. Both frequency and amplitude of a circadian oscillator need to be regulated against fluctuations in order to maintain robust 24-h rhythms [4–6]. In the heart beating, the frequency has to be increased according to the intensity of physical activities [7]. In neuronal firings, proper regulation of the frequency is essential for information transmission in the brain. However, both the heart beating and neuronal firings are seldom required to modulate the amplitude. On the other hand, in glycolytic and cAMP oscillators, the regulation of the amplitude is as important as the regulation of the frequency since the amplitude plays a significant role in the activities of glycolysis and the protein kinase A (PKA) signaling pathway [8, 9].

Thus, how do these oscillators meet such different requirements for regulating the frequency and amplitude? Novak et al. classified biochemical oscillators into three classes: class I oscillators (delayed negative feedback oscillators), class II oscillators (amplified negative feedback oscillators), and class III oscillators (incoherently amplified negative feedback oscillators) [10]. This classification was based solely on the network structure of the oscillator. However, interestingly, the different regulatory requirements seem to be reflected in this classification in view of the fact that (i) the circadian rhythm oscillator belongs to class I oscillators; (ii) the sinus node oscillator and neuronal oscillator belong to class II oscillators; and (iii) the glycolytic and cAMP oscillators fall into class III oscillators. Therefore, a particular type of network structure appears to serve a particular regulatory requirement better than other types, and this implies that there is an association between network structures and regulatory functions.

Such an association between them could also be inferred from the previous study by Tsai et al. in which it was revealed that an interlinked positive and negative feedback structure outperforms a simple negative feedback structure in tuning the frequency of an oscillator [11]. In addition, a positive feedback was revealed to promote the oscillation of a negative feedback oscillator [12]. However, the detailed relationship between various network structures and regulatory functions has only been partially explored till now. To investigate the relationship in detail from a systems perspective, we constructed all possible three-node oscillator models of maximum four links using ordinary differential equations (ODEs) to represent six conceptual network structures of biochemical oscillators, and then performed interaction perturbation to systematically analyze the regulatory pattern of the frequency and amplitude of each model.

So far, the parameter perturbation method has been used to study the properties of oscillators. However, this method might not be adequate to analyze the network-level characteristics of oscillators. Because a parameter can represent various biological functions (e.g., the rate of synthesis or degradation of a molecule, the strength of binding between two molecules and the sensitivity of a reaction), perturbation of a parameter may not correspond to the variation of an interaction in the network. Moreover, the same molecular interaction can be represented in multiple ways (see Additional file 1: Notes). For instance, the interaction ‘X activates Y’ can be represented in several ways:

1 $$ \frac{dY}{dt}={k}_1\cdot X $$

2 $$ \frac{dY}{dt}={k}_1\cdot X\cdot Y $$

and

3 $$ \frac{dY}{dt}=\frac{k_1\cdot X}{K_m+X} $$

In the three equations, the parameter *k*_1_ represents different biological processes, and thus, the perturbation of *k*_1_ will yield various results. In particular, a single parameter can be involved in two interactions (eq. (2) represents two interactions, ‘X on Y’ and ‘Y on Y’), and more than two parameters can represent a single interaction (in eq. (3), *k*_1_ and *K*_*m*_ are involved in the interaction ‘X on Y’). In these cases, the role of an interaction cannot be assessed independently through parameter perturbation analysis. A solution to such limitations could be to modulate the interactions rather than the parameters. For this purpose, we developed a novel perturbation strategy called the interaction perturbation method which directly modulates the strength of an interaction between two nodes in a network. By using this method, we found that a strong association exists between network structures and regulatory patterns of the frequency and amplitude of biochemical oscillators. In simple negative feedback oscillators, both the frequency and amplitude were found to be rarely modulated. In contrast, the frequency could be tuned in amplified negative feedback oscillators while the amplitude could be modulated in incoherently amplified negative feedback oscillators. Our analysis shows that different regulatory properties can emerge from different network structures of biochemical oscillators.

## Results

### Analyses of 3-node biochemical oscillators

**Fig. 1:**
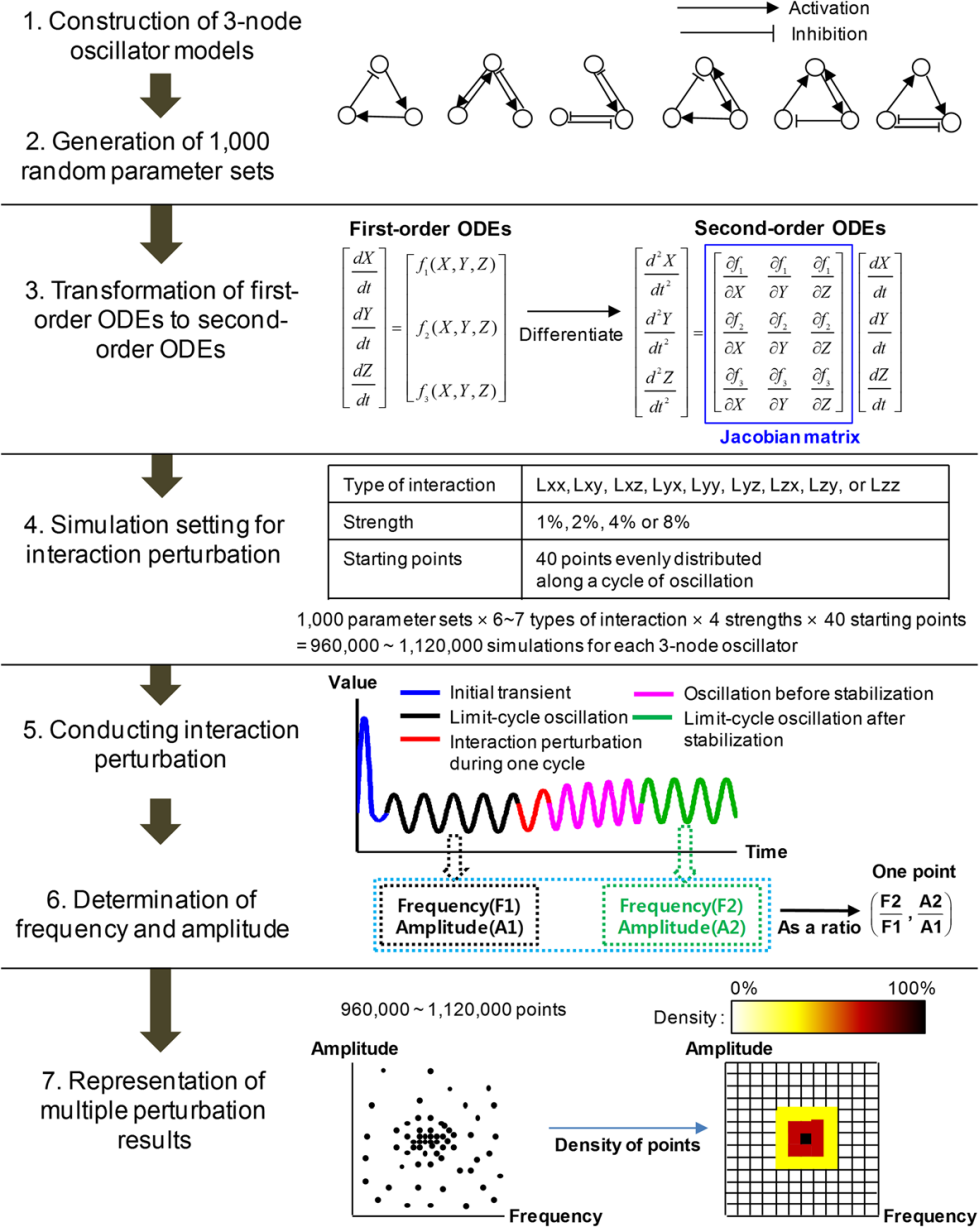
Analysis workflows for three-node oscillator models. **Step 1**. Construct six 3-node oscillator models using first-order ODEs; **Step 2**. Generate 1000 random parameter sets for each ODE; **Step 3**. Reformulate first-order ODEs into second-order ODEs by differentiation with respect to time and locate elements of Jacobian matrix by decomposition of the reformulated second-order ODEs; **Step 4**. Establish the conditions for the perturbations: determine the type of interactions to be perturbed and the strength of the perturbations; **Step 5**. Conduct perturbations under the established conditions; **Step 6**. Measure the resultant frequency and amplitude; and **Step 7**. Create density plots to depict the results schematically

Because biochemical oscillator models are too diverse in their size and complexity to be investigated individually, we constructed all possible representative three-node oscillator models that consist of maximum four links. We began by determining the parameter sets and then conducted analyses of each model based on the interaction perturbation method. The procedures are provided in detail in the METHODS (Fig. 1 and Additional file 1: Figure S1).

#### Network structures of six 3-node biochemical oscillator models

**Fig. 2:**
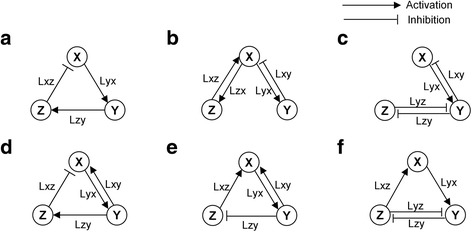
Schematic diagrams of the network structures of the six 3-node biochemical oscillator models. Each model includes three nodes (X, Y, and Z) and nine possible interactions (Lxx, Lxy, Lxz, Lyx, Lyy, Lyz, Lzx, Lzy, and Lzz). The term ‘Lpq’ denotes an interaction in which node P is influenced by node Q. For instance, Lxy denotes an interaction in which node X is influenced by node Y. The figure shows the six network structures: **a** simple NFO; **b** activator-amplified NFO; **c** inhibitor-amplified NFO; **d** type 1 incoherently-amplified NFO; **e** type 2 incoherently-amplified NFO; and (**f**) type 3 incoherently-amplified NFO in order of appearance

Each model includes three nodes (X, Y, and Z) and nine possible types of interactions (Lxx, Lxy, Lxz, Lyx, Lyy, Lyz, Lzx, Lzy, and Lzz) (Fig. 2). In all six models, the term X, Y, and Z denote the ‘activator’, ‘inhibitor’, and ‘mediator’, respectively, that is, X activates Y; Y inhibits X, and Z mediates the activation or inhibition.

The simple negative feedback oscillator (simple NFO) has a negative feedback loop only (Fig. 2a). For the simple NFO to be able to oscillate, at least three nodes have to be included in its negative feedback loop since the time delay required for the sustenance of the oscillation cannot be sufficiently provided with only two nodes. Adding a third node (denoted by Z in Fig. 2a) generates an appropriate time delay. In this structure, Lyx, Lzy, and Lxz form a negative feedback loop where X activates Y directly, and Y inhibits X through Z, which is consistent with the denoted function of X and Y: X as ‘activator’, and Y as ‘inhibitor’. In the activator-amplified negative feedback oscillator (activator-amplified NFO), X and Y form a two-node negative feedback loop (Lxy and Lyx), and Z amplifies X (Lxz and Lzx) (Fig. 2b). This amplification by Z plays an important role in maintaining oscillation [13]. In the inhibitor-amplified negative feedback oscillator (inhibitor-amplified NFO), X and Y form a two-node negative feedback loop (Lxy and Lyx), and Z amplifies Y (Lyz and Lzy) (Fig. 2c). Like the activator-amplified NFO, the amplification by Z is essential for the maintenance of oscillation [13]. Each incoherently-amplified negative feedback oscillator (incoherently-amplified NFO) has a negative feedback loop containing one incoherent link. We constructed three incoherently-amplified NFOs: type 1 incoherently-amplified NFO; type 2 incoherently-amplified NFO; and type 3 incoherently-amplified NFO (Fig. 2d, e, and f). Among all the types of incoherently-amplified NFOs, Lyx, Lzy and Lxz form a negative feedback loop containing an incoherent link. An incoherent link makes the function of a node become inconsistent with its denoted function as an activator or inhibitor. For instance, in the type 1 incoherently-amplified NFO, Y inhibits X via Z (Lzy and Lxz) and directly activates X (Lxy) simultaneously. Thus, Y is no longer an ‘inhibitor’. The incoherent link is Lxy in the type 1 and type 2 incoherently-amplified NFO and Lyz in the type 3 incoherently-amplified NFO.

According to the classification by Novak et al., the above six models can be classified into three classes: the simple NFO belongs to class I oscillators; the activator-amplified NFO and the inhibitor-amplified NFO belong to class II oscillators; and all types of incoherently-amplified NFOs belong to class III oscillators [10].

#### Analysis results of the six 3-node oscillation models

**Fig. 3:**
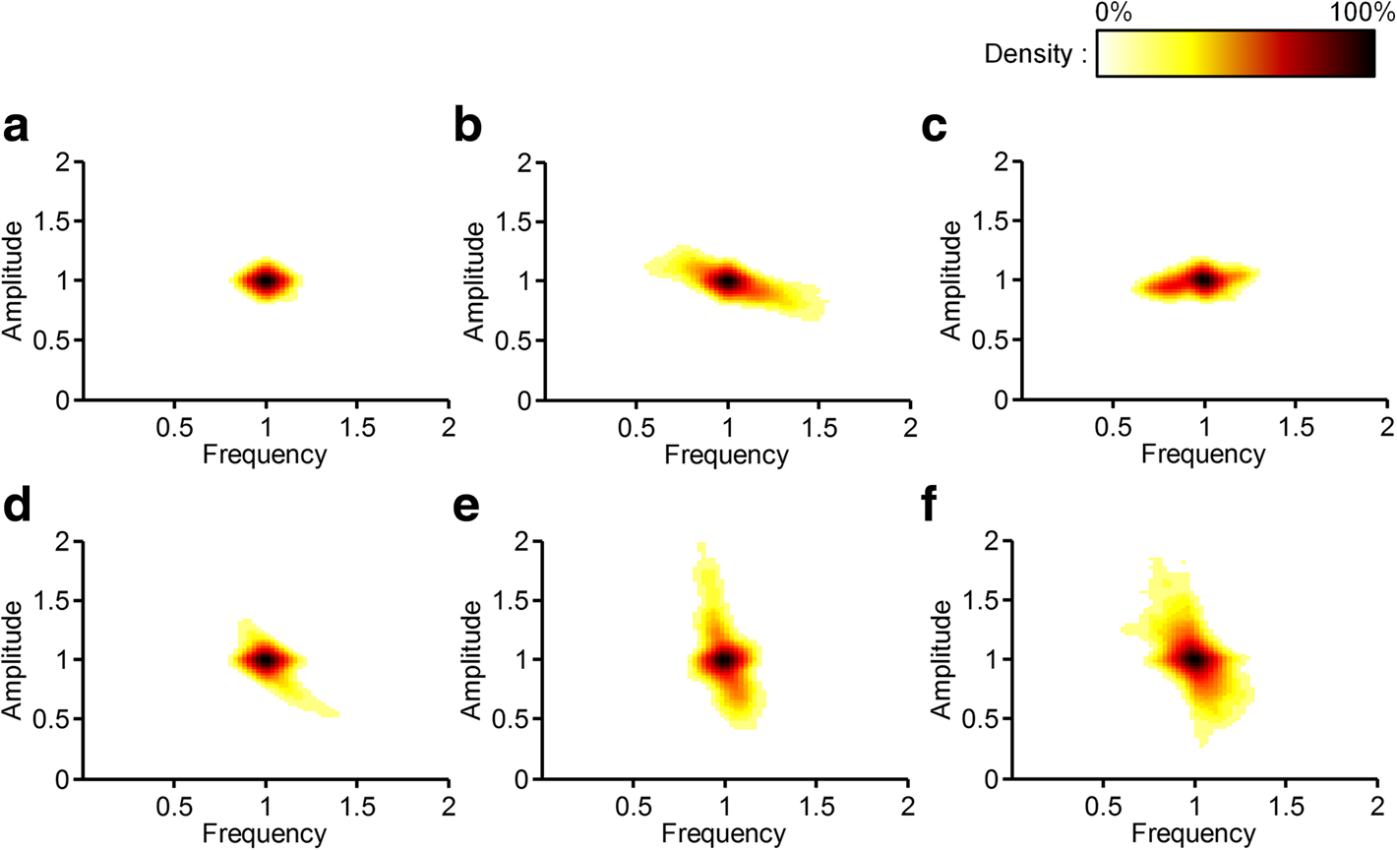
The density plots of the six 3-node oscillator models. In these plots, the regulatory characteristics of the frequency and amplitude are represented by the distribution patterns of the density. The density is increasing over a continuum starting from white followed by yellow, red, and black. This figure shows the six network structures: **a** simple NFO; **b** activator-amplified NFO; **c** inhibitor-amplified NFO; **d** type 1 incoherently-amplified NFO; **e** type 2 incoherently-amplified NFO; and (**f**) type 3 incoherently-amplified NFO in order of appearance

To investigate the regulatory patterns of the three-node oscillators, we performed perturbations by weakening each interaction by 1%, 2%, 4%, and 8% (a weakening factor of 0.99, 0.98, 0.96, and 0.92, respectively) and observed the changes in the frequency and amplitude. The interaction perturbation was implemented by multiplying a weakening factor to the element of Jacobian matrix that is to be perturbed during one period of oscillation. Fig. 3 shows the results of the perturbations in the density plots (see the METHODS for details). In these plots, the regulatory characteristics of the frequency and amplitude are represented by the distribution patterns of the density. The concentrated density near the point (1, 1) indicates that the frequency and amplitude are robust to perturbations. The horizontal distribution of the density denotes that the change in the frequency is larger than the change in the amplitude, and the vertical distribution of the density denotes the opposite.

To represent the results quantitatively, we grouped the changes in the frequency and amplitude into three patterns: In pattern R, both the frequency and amplitude changed by less than 1%; in pattern F, either the frequency or the amplitude changed by more than 1% and the changes in the frequency were greater than the changes in the amplitude; in pattern A, either the frequency or the amplitude changed by more than 1% and the changes in the amplitude were greater than the changes in the frequency. Table 1 shows the distribution of the patterns in each 3-node oscillator model (a full description of the distribution of the patterns generated by the perturbation of each interaction is provided in Additional file 1: Table S1).

**Table 1:** Regulatory patterns of the frequency and amplitude arising from interaction perturbation

Network structure of oscillators	Distribution of patterns (%)		
	Pattern R	Pattern F	Pattern A
Simple NFO	92.9%	0.7%	6.4%
Activator-amplified NFO	61.5%	32.3%	6.2%
Inhibitor-amplified NFO	34.1%	43.6%	22.3%
Type 1 incoherently-amplified NFO	72.2%	10.1%	17.7%
Type 2 incoherently-amplified NFO	39.5%	5.5%	55.0%
Type 3 incoherently-amplified NFO	16.9%	27.0%	56.1%

The simple NFO showed the highest robustness to perturbations regardless of the types of perturbed interactions (Fig. 3a and Additional file 1: Figure S2). The rates of change in both the frequency and amplitude were less than 1% in 92.9% of the perturbation results (Table 1) and are depicted as the darkest density concentrated on the point (1, 1) (Fig. 3a).

In the activator-amplified NFO and inhibitor-amplified NFO, the change in the frequency was larger than the change in the amplitude. The results are shown in Fig. 3b and c, in which the density is spread in a nearly horizontal direction. These changes in the frequency were caused by the perturbations of Lxx or Lxy (Additional file 1: Figure S3 and Additional file 1: Figure S4). In both oscillators, pattern F was observed in more than 30% of the perturbation results.

In contrast to the regulatory patterns observed in the activator-amplified NFO and the inhibitor- amplified NFO, all the incoherently-amplified NFOs showed moderate changes in the amplitude. In the type 1 incoherently-amplified NFO, the amplitude was slightly more adjustable to perturbations than the frequency while in the type 2 incoherently-amplified NFO, the amplitude was changed to a large extent (Fig. 3d and e). In the type 3 incoherently-amplified NFO, the frequency and amplitude were changed to various extents (Fig. 3f). Both in the type 2 incoherently-amplified NFO and the type 3 incoherently-amplified NFO, pattern A was observed in more than 50% of the perturbation results.

For the six 3-node oscillator models, the perturbation results obtained by weakening each interaction by 2%, 4%, or 8% had qualitatively the same regulatory patterns as those obtained by weakening each interaction by 1% (Additional file 1: Figures S2-S7).

In summary, a distinct regulatory pattern was observed in each 3-node oscillator. Class I oscillator (the simple NFO) is robust to perturbations while for class II oscillators (the activator-amplified NFO and the inhibitor-amplified NFO), the frequency can be selectively regulated. In class III oscillators (types 1, 2, and 3 incoherently-amplified NFOs), the amplitude can be regulated. Based on these observations, we deduced the regulatory principle that the differences in network structures give rise to different regulatory patterns of the frequency and amplitude.

### Mathematically controlled comparisons between structurally related biochemical oscillators

Class I, class II and class III oscillators are structurally related to one another, and their structural differences arise from an added link. A class II oscillator can be formed by adding a link to the activator (X) or inhibitor (Y) of a class I oscillator. A class III oscillator can be formed by adding an incoherent link to a class I oscillator.

This prompted us to assume that the added links could bring about changes to the regulatory patterns of the oscillators. To examine this idea, we performed mathematically controlled comparisons between structurally related oscillators.

#### Construction of structurally related three-node models for mathematically controlled comparisons

We developed three additional three-node oscillator models which contain one additional link to the backbone of a simple NFO. A self-activating positive feedback link was added to the activator (X) and inhibitor (Y) of the simple NFO to generate an activator-amplified NFO variant and an inhibitor-amplified NFO variant, both of which belong to class II oscillators. Adding Lxy to the simple NFO generated a variant of the type 1 incoherently-amplified NFO (hereinafter called the type 1 incoherently-amplified NFO variant), which belongs to class III oscillators. Simulation of the simple NFO was performed with a representative parameter set suggested by Novak et al. [10]. The parameters of the newly generated oscillators (the activator-amplified NFO variant, the inhibitor-amplified NFO variant and the type 1 incoherently-amplified NFO variant) were determined with methods for mathematically controlled comparisons [14]. The full ODEs are provided in Additional file 1: Eq. A1, and the full parameters are provided in Additional file 1: Table S3.

#### Analysis results of the structurally related models

**Fig. 4:**
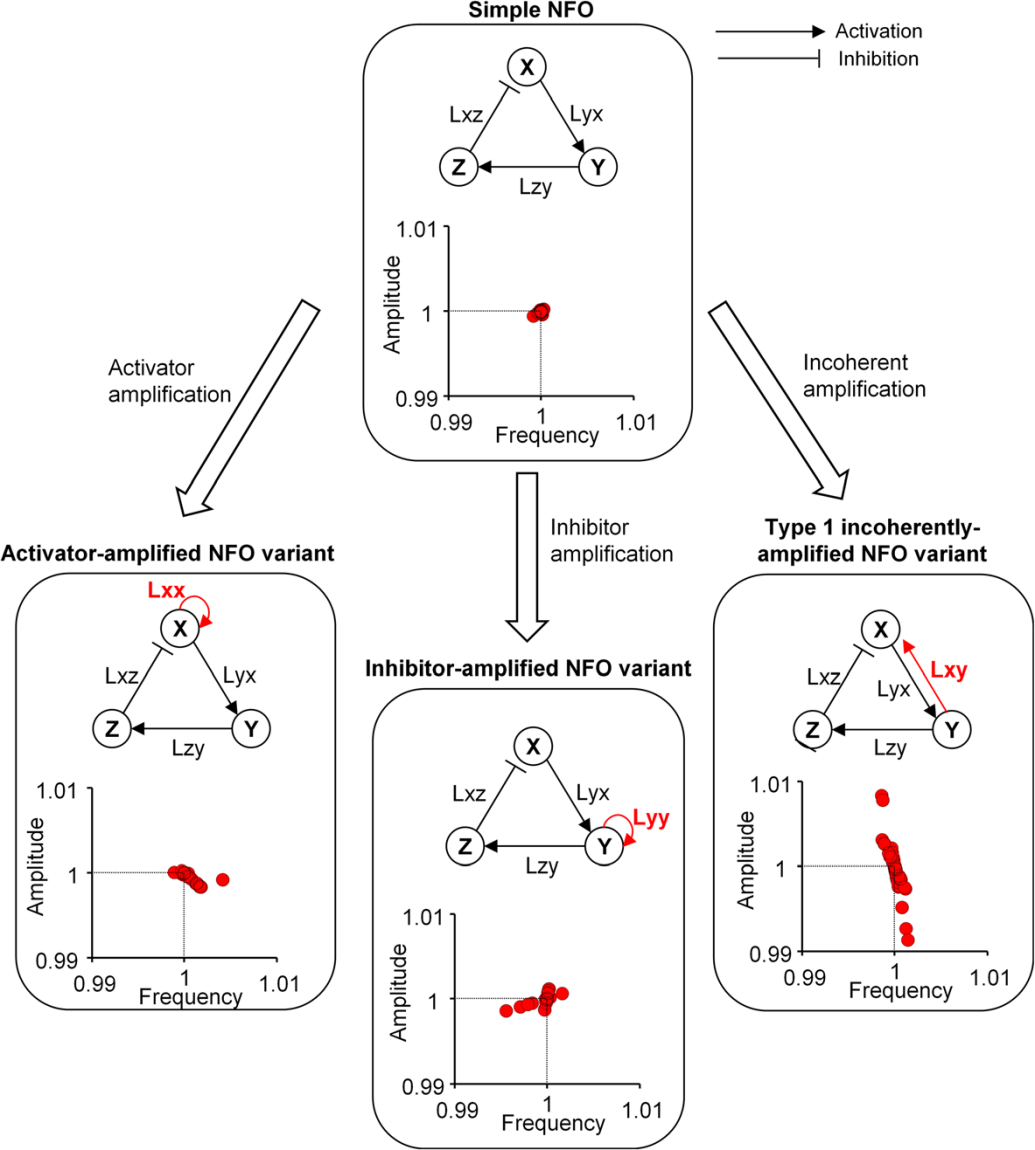
Mathematically controlled comparisons among the simple NFO, the activator-amplified NFO variant, the inhibitor-amplified NFO variant, and the type 1 incoherently-amplified NFO variant. Schematic representations and frequency-amplitude plots for the four oscillator models are presented for comparison. In these plots, the changes in the frequency and amplitude due to the perturbations are expressed as a ratio to the frequency and amplitude before the perturbations

Perturbations on the oscillators were performed by weakening each interaction by 1% during one period of oscillation. A distinct regulatory pattern for each model could be identified despite the fact that the frequency and amplitude changed by less than 1% compared to the unperturbed cases in all four models (Fig. 4). In the activator-amplified NFO variant and the inhibitor-amplified NFO variant, the frequency was more adjustable than the amplitude, whereas in the type 1 incoherently-amplified NFO variant, the amplitude was more adjustable than the frequency. Overall, adding an amplifying link could enhance the ability to regulate the frequency of the oscillator whereas adding an incoherent link could enhance the ability to regulate the amplitude of the oscillator.

### Analyses of naturally-occurring biochemical oscillator models

**Fig. 5:**
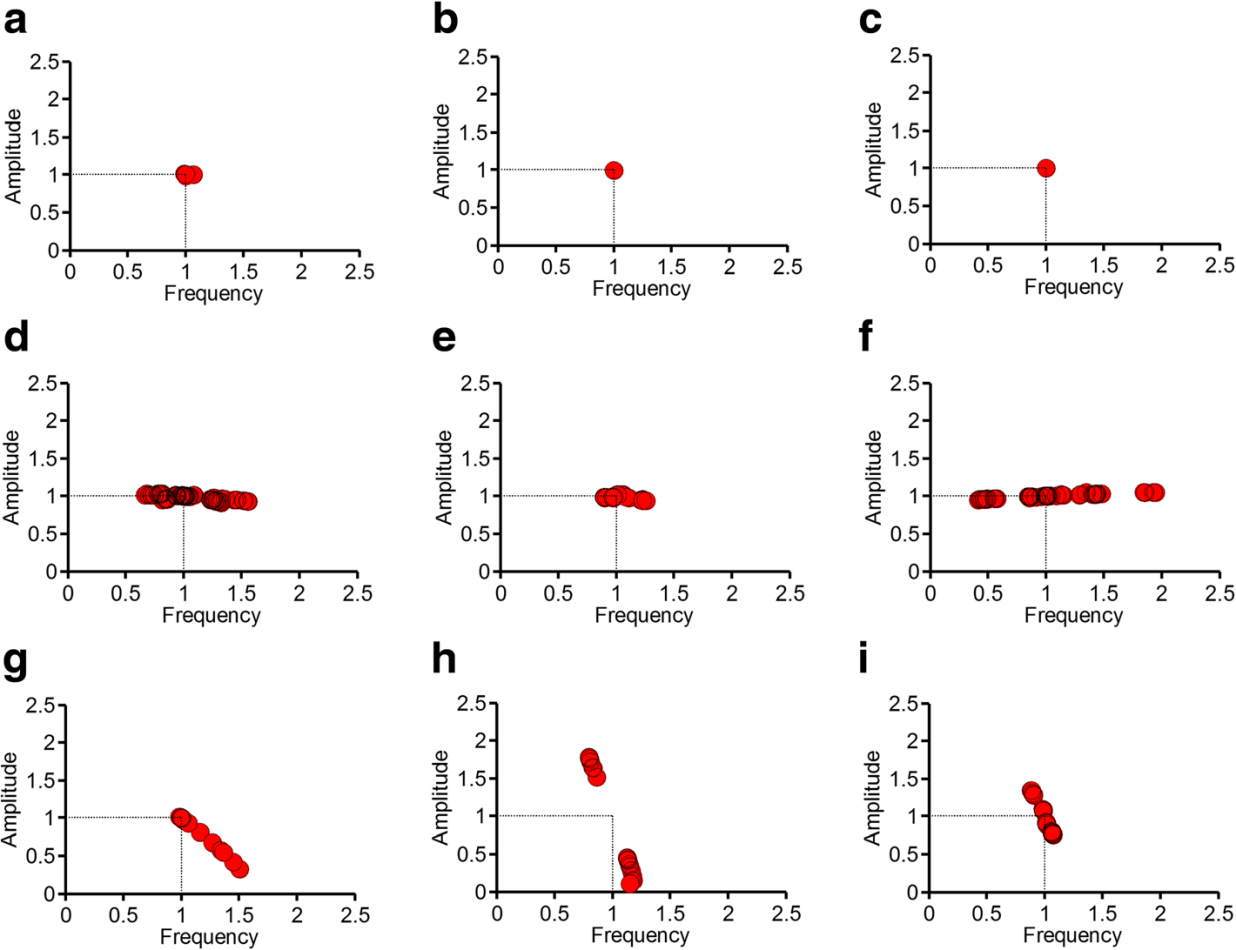
Analyses of naturally-occurring biochemical oscillator models. The changes in the frequency and amplitude are represented in the frequency-amplitude plots for the following: **a** circadian rhythm model by Leloup et al.; **b** circadian rhythm model by Goldbeter; **c** repressilator by Elowitz and Leibler; **d** sinus node model by Yanagihara et al.; **e** neuronal model by Hodgkin and Huxley; **f** cell cycle model by Pomerening et al.; **g** cAMP model by Martiel and Goldbeter; **h** glycolysis model by Sel’kov; and (**i**) glycolysis model by Higgins. In these plots, the changes in the frequency and amplitude due to perturbations are expressed as a ratio to the frequency and amplitude before the perturbations

To explore whether the regulatory principle suggested here could also apply to naturally-occurring biochemical oscillator models, we performed analyses of nine well-known biochemical oscillator models which were constructed based on experimental data. For each model, perturbations were conducted by weakening each interaction by 1% during one-period of oscillation. The subsequent changes in the frequency and amplitude are shown in Fig. 5. The ODE equations and the parameters are provided in the Additional file 1: Eq. A2.

The circadian rhythm model by Goldbeter [15], the circadian rhythm model by Leloup et al. [16] and the repressilator [17] are well-known examples of class I oscillators (a class I oscillator consists of a negative feedback only)**.** These oscillators showed the highest robustness to perturbations: both the frequency and amplitude rarely changed in response to the perturbations. The sinus node model by Yanagihara et al. [18], the neuronal excitation model by Hodgkin and Huxley [19] and the cell cycle model by Pomerening et al. [20] can be classified as class II oscillators (a class II oscillator includes a positive feedback). These class II oscillators worked better in adjusting the frequency than in adjusting the amplitude; the perturbations induced more changes to the frequency than to the amplitude. On the other hand, in the cAMP oscillator model [21] and the glycolysis models [22, 23] that belong to class III oscillators (a class III oscillator includes an incoherent link), the amplitude was more adjustable than the frequency: the amplitude changed more than the frequency.

In summary, the regulatory principle suggested here in the three-node oscillator models could also apply to naturally-occurring biochemical oscillator models.

## Discussion

Our analysis based on the interaction perturbation method revealed the regulatory principle that different network structures of biochemical oscillators give rise to different regulatory patterns of the frequency and amplitude; for class I oscillators, the frequency and amplitude are seldom regulated; for class II oscillators, the frequency is more adjustable than the amplitude; for class III oscillators, the amplitude is more adjustable than the frequency. The results of the mathematically controlled comparisons further demonstrated the reliability of this regulatory principle and its potential for application to naturally-occurring biochemical oscillator models.

In systems biological studies, the parameter perturbation method has been widely used to investigate the relationship between network structures and their biological functions [24–29]. In addition, various mathematical methods have been developed to analyze the characteristics of oscillators. The sensitivity heat map and parameter sensitivity spectrum developed by Rand et al. have been utilized to provide a more integrated picture of the overall sensitivities of a system and to probe how the function of a network depends upon its structure and parameters [30]. Irene et al. proposed an optimization-based approach to investigate what environmental conditions drive specific oscillatory network [31]. The state sensitivity decomposition method developed by Wilkins et al. is useful in analyzing the influence of parameter changes on period, amplitude and relative phase of oscillation [32]. In addition, robustness and dynamical characteristics between various oscillatory systems could be effectively compared by using bifurcation analysis, parameter sensitivity analysis, and stochastic simulation [33, 34]. However, since all these methods somehow focus on “parameters”, they can be classified as a parameter perturbation analysis in a broad sense. On the other hand, as far as the network topology is concerned, the biological meaning of a parameter does not always correspond to specific interaction. Hence, there is still difficulty in attributing the perturbation of a particular interaction in a regulatory network to the perturbation of a parameter in the corresponding mathematical model.

The interaction perturbation method proposed in this study has several advantages over the parameter perturbation method. First, the result of an interaction perturbation analysis can be properly interpreted in the context of a biological network since the perturbation directly modulates a link of the network structure. Second, this method can provide a more pertinent comparison between different network structures by allowing the focus of the comparison to be placed on the difference of the interaction in the network, not on the indirect difference of the underlying biological process. If the network structures have the same number of nodes, the comparison can be performed more effectively between them as they have a Jacobian matrix of the same size (three-node networks have a Jacobian matrix of 3 by 3). Third, this method can simplify analysis procedures. Previously suggested methods (e.g., optimization-based method, state sensitivity decomposition method, *etc*) for the analysis of biochemical oscillators can provide meaningful insights into the nature of oscillators, but most of them require a certain level of expert knowledge on mathematics [31, 32]. In contrast, with a given parameter set, we just need to transform first-order ODEs into second-order ODEs and integrate the second-order ODEs using a perturbed Jacobian matrix without going through any other complicated procedures such as selection of a bifurcation parameter, identification of a Hopf bifurcation point and numerical continuation [35].

In this study, we demonstrated that the regulatory patterns of the frequency and amplitude depend on the network structures of the biochemical oscillators. Notably, even for the same class of network structures, different regulatory patterns were observed. For instance, for the activator-amplified NFO, the amplitude was adjustable although the range was narrow whereas for the inhibitor-amplified NFO, modulation of the amplitude was negligible. The regulatory range of the amplitude was wider for the type 2 incoherently-amplified NFO than that for the type 1 or a type 3 incoherently-amplified NFO. For the type 3 incoherently-amplified NFO, both the amplitude and frequency could be regulated to a various extent. Thus, not only overall network topologies but the interlinkage of nodes appear to be involved in the formation of regulatory patterns of the frequency and amplitude.

It may also be noteworthy to mention that, in this study, the chosen parameter set and the kind of interactions were identified as a minor contributory factor that could affect the regulatory patterns of the frequency and amplitude, though not significantly. For class I oscillators, the frequency and amplitude were changed by less than 1% for most of the parameter sets (pattern R) except for a few parameter sets where the amplitude was changed by more than 1% (pattern A). For class II oscillators, the frequency was adjustable for more than one third of the parameter sets (pattern F) whereas, for the others, the frequency was not changed (patterns R and A). For class III oscillators, the amplitude was adjustable for a relatively greater part of the parameter sets (pattern A) whereas, for the others, the amplitude was not changed (patterns R and F).

For the activator-amplified NFO and inhibitor-amplified NFO, by perturbation of Lxx or Lxy, the frequency was adjusted whereas the perturbations did not significantly change the frequency or amplitude. In the incoherently amplified NFOs, only some kinds of interactions seem to be involved in modulation of the amplitude.

Our analyses of naturally-occurring biochemical oscillator models showed that the regulatory principle suggested here may have applications in naturally-occurring biochemical oscillation models. A question then might arise as to what functional benefits can be derived from a particular network structure in naturally-occurring biochemical oscillators? Both frequency and amplitude of a circadian oscillator need to be regulated against fluctuations in order to maintain robust 24-h rhythms [36, 37]. This requirement can be satisfied by the network structure of a class I oscillator. When an incoherent feedforward structure is added to such a circadian oscillator, a stable oscillator with a different frequency can be generated and used to meet other biological needs [38]. Sinus nodal cells and neurons should be able to tune their frequency to transmit the information to neighboring cells appropriately [39] and cell cycles have to regulate the rate of their progression appropriately in response to environmental changes. To this end, the network structure of a class II oscillator might be a suitable one. In the glycolysis and cAMP models, regulation of the amplitude has greater importance since the amplitude of phosphofructokinase and cAMP, which are actively involved in metabolic and signaling processes respectively, has a significant role in cellular functions. Therefore, the network structure of a class III oscillator can be a better choice for these cases.

From an evolutionary point of view, the fact that class II and class III oscillators are frequently found in natural biological examples can be considered as important evidence implicating that they might have some performance advantages over class I oscillators. This study suggests that such advantages might be found from the ability to tune the frequency in class II oscillators and the ability to regulate the amplitude in class III oscillators.

Our study has the following limitations. Firstly, our study employed the Jacobian matrix which describes the interactions between state variables instead of employing the monodromy matrix (i.e., the state transition matrix over one period) or Floquet multipliers (i.e., eigenvalues of the monodromy matrix) which have been widely used to determine the oscillatory properties of a system with a limit cycle. Therefore, our scope of analysis was largely confined to examining influences of interaction perturbation on the oscillatory properties. Secondly, interaction perturbation was performed during one cycle of oscillation because longer duration of perturbation destabilized the oscillation in most cases. Thirdly, our analysis of structurally related models may not be sufficient to investigate the general characteristics of each structure in greater detail since it was performed under the pre-defined parameter combinations.

## Conclusions

From the analyses based on the interaction perturbation method, we found a new regulatory principle that differences in network structures can give rise to different regulatory patterns of the frequency and amplitude. This finding could serve as a basis for further investigation into the underlying mechanism for the regulation of the frequency and amplitude in existing biochemical oscillators as well as for designing synthetic oscillators with a specific regulatory function.

## Methods

### Analysis procedures for 3-node biochemical oscillators

We constructed six representative oscillator models and generated random parameter sets for each model ensuring its sustained oscillation under the parameter sets. Then, we converted the interactions in the model into corresponding elements of the Jacobian matrix and performed perturbations of the elements. After the perturbations, we measured resulting changes of the frequency and amplitude. The analysis workflows are described in Fig. 1 and Additional file 1: Figure S1.

#### Construction of six representative models for biochemical oscillators

Each three-node oscillator model was described in terms of three coupled ODEs with the combinatorial use of mass action laws and Michaelis-Menten kinetics. The ODEs of the six 3-node oscillator models are provided in the Additional file 1: Eq. A1. In every oscillator model, the oscillations were sustained under specific parameter sets. After an initial transient, integrations that started under different initial conditions quickly converged to a common trajectory with the same frequency and amplitude, namely, a limit-cycle oscillation.

#### Random parameter generation for the six 3-node biochemical oscillator models

Determining the parameter values constitutes an important process to create sustained oscillations. We chose to determine parameter values by extensive search of the parameter space because an analytical approach does not lend itself to dealing with a large number of parameters.

Random parameter sets were generated for each three-node oscillator model by selecting parameters from an exponential distribution within the range of 0.001 to 1000, using the Latin hypercube sampling method [40]. This range corresponds to biologically reasonable values typically used to model biological systems [41–45]. All parameters except for the Hill coefficients were randomly generated. For each parameter set, we verified whether the model produced a limit-cycle oscillation [46]. Through this process, a total of 1000 parameter sets were generated for each model, and consequently, each three-node oscillator model yielded 1000 parameter-value-assigned models.

#### Algebraic representation of interaction using Jacobian matrix

A network topology shows clearly whether an interaction is activating or inhibiting. However, when this network is represented by the system of ODEs, the function of interaction is not easily identifiable. To represent an interaction as an algebraic object, the Jacobian matrix will be a reasonable choice since an element of the Jacobian matrix corresponds to an interaction. Because an element of the Jacobian matrix cannot be obtained directly from first-order ODEs, we differentiated the first-order ODEs with respect to time to generate second-order ODE systems (step 3 in Fig. 1). Thus, this system can be represented by the matrix product of the Jacobian matrix and first-order ODEs. Here, the Jacobian matrix is defined as *a*_*ij*_ = ∂*f*_*i*_/∂*x*_*j*_, where *i* and j denote the row and column indices of the Jacobian matrix, respectively. A non-zero value of *a*_*ij*_means that variable *x*_*j*_ influences the evolution of variable *x*_*i*_; in other words, an interaction from node *j* to node *i* exists [47].

First-order ODEs were numerically integrated using various initial conditions until they converged to a limit-cycle oscillation. In order to confirm that second-order ODEs are good approximations to first-order ODEs, we simulated second-order ODEs and first-order ODEs using a point in a limit-cycle oscillation as an initial point. The results showed almost no differences (see Additional file 1: Table S2 for the mean differences between the two simulations).

#### Perturbation conditions

Because the aim of this study is to investigate the difference in the regulatory patterns of the frequency and amplitude between network structures, the following perturbation conditions for each parameter-value-assigned model were set so that the regulatory patterns should not be influenced by perturbation conditions: the type of interaction to be perturbed; the perturbation strength; the perturbation duration; and the perturbation starting point.

Every type of interaction was perturbed one by one since the function of an interaction may not be distinguishable if more than two interactions were perturbed simultaneously. The strength of the perturbations was weakened by 1%, 2%, 4%, and 8%, which correspond to weakening factors of 0.99, 0.98, 0.96, and 0.92, respectively. These weakening degrees were selected because weakening perturbations by more than 8% often destabilized the limit cycle oscillation.

After determining the type of interactions and weakening factors, we multiplied a weakening factor by the corresponding element of the Jacobian matrix to construct the Jacobian matrix of the perturbation. For instance, when we perturbed Lyx with a weakening factor of 0.99, we multiplied 0.99 to the element at the second row and first column of the Jacobian matrix leaving all other remaining elements the same.

The perturbations were performed during one period of oscillation since the results could vary according to the oscillatory phases. We established 40 starting points of perturbations which were evenly distributed along the time cycle to prevent the trajectories from being influenced by the positions where the perturbations began.

#### Perturbation processes

A first-order ODE was numerically integrated using various initial conditions until it reached a starting point of perturbation. We simulated a second-order ODE using the perturbed Jacobian matrix during one period of oscillation. After that, the second-order ODE was integrated using the unperturbed Jacobian matrix until the oscillation was stabilized.

#### Representation of perturbation results

We measured the frequency and amplitude when the oscillation was stabilized after completion of a perturbation while excluding the case of damped oscillations. The percentages of the parameter sets out of total parameter sets for 3-node oscillators that did not show sustained limit-cycle oscillations are provided in Additional file 1: Table S4. The change in the frequency and amplitude is presented as a ratio to the frequency and amplitude of the oscillation before the perturbation. For instance, the ratio (2, 0.5) means that the frequency doubled and the amplitude halved.

The perturbations of a three-node oscillator model generated around 1000,000 results. To make the results more intuitive and easily understood, we depicted them in density plots. We divided the whole frequency-amplitude domain into 10,000 equal-sized sub-domains, and calculated the number of results that belong to each sub-domain and the percentage that it occupies in the total number of results, and then converted the calculated percentage into density and each color-coded sub-domain according to its density; in the plots, the density is increasing over a continuum starting from white followed by yellow, red and black.

### Mathematically controlled comparisons

To determine the parameter sets for the activator-amplified NFO variant, the inhibitor-amplified NFO variant, and the type 1 incoherently-amplified NFO variant, we adopted a method for mathematically controlled comparisons proposed by Michael A. Savageau [14]. First, the values of the parameters for the unaltered processes of one system are assumed to be identical with those of the corresponding parameters of the other system. For instance, in the activator-amplified NFO variant, degradation of X, synthesis of Y, degradation of Y, synthesis of Z, and degradation of Z are unaltered processes in comparison to the simple NFO. So, parameters for those processes (k_dx_, k_1_, k_2_, K_m_, and k_3_) in the activator-amplified NFO variant are equal to the corresponding parameters of the simple NFO. Second, parameters associated with altered processes are free to assume any values. In the activator-amplified NFO variant, parameters related with the synthesis of X are assumed to have any values. Third, the parameters of the altered processes are determined by imposing constraints on the external behavior of the system. For the above three oscillation models, the following two constraints were imposed to determine the free parameters: (i) integration of the ODE models with the specified parameter sets have to be able to generate a limit-cycle oscillation; (ii) the frequency and amplitude of the oscillation have to be similar to those of the simple NFO.

After determination of the parameters, perturbations were performed in the same manner as in the conceptual three-node models. Then, the changes in the frequency and amplitude in the four oscillator models (the simple NFO, the activator-amplified NFO variant, the inhibitor-amplified NFO variant, and the type 1 incoherently-amplified NFO variant) were compared.

## Additional file

Additional file 1: Details on methods and results of the interaction perturbation analysis for comprehensive assessment of the regulatory principle underlying various biochemical oscillators. (PDF 3894 kb)

## Abbreviations

cAMP: cyclic adenosine monophosphate
MAPK: mitogen-activated protein kinase
NFO: negative feedback oscillator
ODE: ordinary differential equation
PKA: protein kinase A
