# Supplementary material for: A novel interaction perturbation analysis reveals a comprehensive regulatory principle underlying various biochemical oscillators
Source: BMC Syst Biol. 2017 Oct 10;11:95. doi: 10.1186/s12918-017-0472-7 (PMC5635494; doi:10.1186/s12918-017-0472-7)
Supplement: Additional file 1: — Details on methods and results of the interaction perturbation analysis for comprehensive assessment of the regulatory principle underlying various biochemical oscillators. (PDF 3894 kb) [file 12918_2017_472_MOESM1_ESM.pdf]

A novel interaction perturbation analysis reveals a comprehensive regulatory principle underlying various biochemical oscillators

Jun Hyuk Kang[1,2] and Kwang-Hyun Cho[1,2‡]

[1]Laboratory for Systems Biology and Bio-inspired Engineering, Department of Bio and Brain Engineering, Korea Advanced Institute of Science and Technology (KAIST), Daejeon, 34141, Republic of Korea

[2]Graduate School of Medical Science andin Engineering, Korea Advanced Institute of Science and Technology (KAIST), Daejeon, 34141, Republic of Korea

Additional Information

‡Corresponding author. E-mail: ckh@kaist.ac.kr, Phone: +82-42-350-4325, Fax: +82-42-350-4310, Web: http://sbie.kaist.ac.kr/

Additional Information

Table of Contents

I. Additional Figures

Additional Figure S1. Step-by-step procedures for the interaction perturbation method to analyze an activator-amplified NFO.

Additional Figure S2. Density plots of the simple NFO.

Additional Figure S3. Density plots of the activator-amplified NFO.

Additional Figure S4. Density plots of the inhibitor-amplified NFO.

Additional Figure S5. Density plots of the type 1 incoherently-amplified NFO.

Additional Figure S6. Density plots of the type 2 incoherently-amplified NFO.

Additional Figure S7. Density plots of the type 3 incoherently-amplified NFO.

II. Additional Tables

Additional Table S1. Regulatory patterns of the frequency and amplitude according to the types of interactions

Additional Table S2. Relative mean differences between 1st-order ODE and 2nd-order ODE in the results of the simulations during ten periods of oscillations.

Additional Table S3. Parameter sets for the 3-node biochemical oscillator models for mathematically controlled comparison

Additional Table S4. Percentage of parameter sets for 3-node biochemical oscillator models that did not sustain limit-cycle oscillation after interaction perturbation from total parameter sets.

III. Additional Equations

Additional Equation A1. ODEs of the 3-node biochemical oscillators

Additional Equation A2. ODEs and parameters for naturally occurring biochemical oscillator models.

IV. Additional Notes

Additional Note A1. Advantages of interaction perturbation over parameter perturbation in this study

Additional Note A2. Difference between 1st-order ODE and 2nd-order ODE in respect of responses to a

perturbation of the system

I. Additional Figures

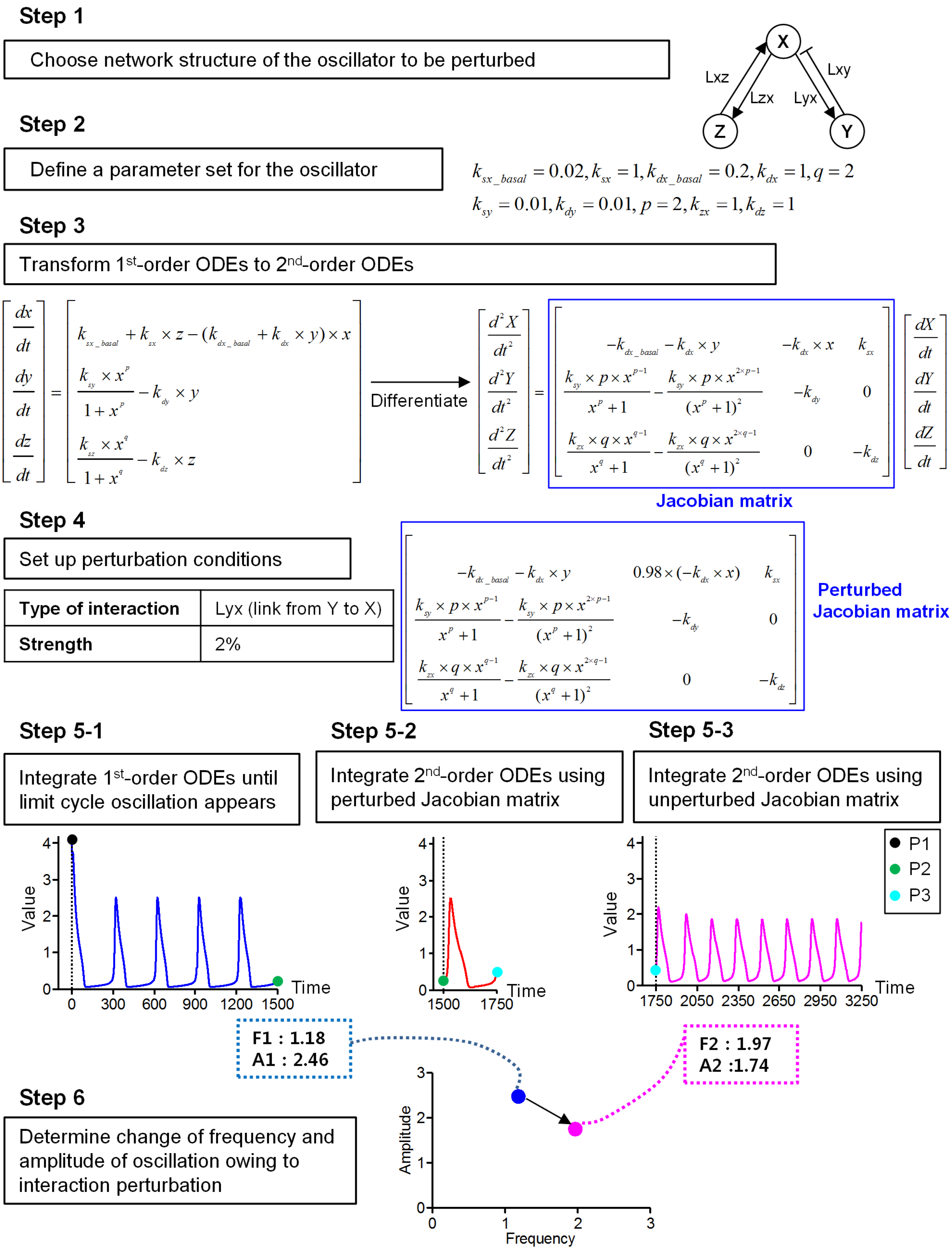

Additional Figure A1. Step-by-step procedures for the interaction perturbation method to analyze an activator-amplified NFO. The steps in Figure A1 are an example of the steps in Figure 1. Step 1. An activator-amplified NFO is chosen for interaction perturbation; Step 2. A parameter set is defined so that a limit cycle oscillation can be generated; Step 3. The first-order ODEs are transformed into the equivalent second-order ODEs by differentiation. The second-order ODEs are represented by the matrix product of the Jacobian matrix and first-order ODEs. Here, the Jacobian matrix shows its complete algebraic form. The element of the Jacobian matrix at the intersection of the ith row and j^{th} column denotes the interaction from node j to node i. For example, the expression, -kdx×x, corresponds to the first row and the second column element of the Jacobian matrix. Therefore, it denotes the interaction from Y to X); Step 4. The interaction from Y to X is weakened by 2% during one period of oscillation. The weakening factor of 0.98 is applied to the expression, -kdx×x, for this perturbation; Step 5-1. The first-order ODEs are numerically integrated until a limit cycle oscillation is generated. The time course of the variable X is plotted. The point P1 $((X(t=t_0), Y(t=t_0), Z(t=t_0)) = (4,0,0))$ indicates the initial values for this integration; Step 5-2. A point is taken along a limit cycle (i.e., P2), and then the second-order ODEs are integrated using the perturbed Jacobian matrix during one period of oscillation. The point P2 is

$(X(t=t_{1500}), Y(t=t_{1500}), Z(t=t_{1500}), \frac{dX}{dt}(t=t_{1500}), \frac{dY}{dt}(t=t_{1500}), \frac{dZ}{dt}(t=t_{1500}))$ = (0.1995, 0.0582, 0.0366, 0.0051, -0.0002, 0.0007). Note that the values (i.e., X, Y, Z) as well as the time derivatives (i.e., dXdt, dYdt, dZdt) of the state variables are required for integration of the second-order ODEs; Step 5-3. After the perturbation (at point P3), the second-order ODEs are integrated using unperturbed Jacobian matrix. The point P3 is $(X(t=t_2), Y(t=t_{1750}), Z(t=t_{1750}), \frac{dX}{dt}(t=t_{1750}), \frac{dY}{dt}(t=t_{1750}), \frac{dZ}{dt}(t=t_{1750}))$ = (0.5066, 0.1111, 0,1777, 0.0506, 0.0009, 0.0266). Although the second-order ODEs are integrated using the

parameter sets which are the same as those for the integration of the first-order ODEs, the different trajectories are obtained because of the different subsets of the initial conditions (i.e., in the case of the first order ODEs: X, Y, Z; in the case of the second order ODEs: X, Y, Z, dXdt, dYdt, dZdt); Step 6. The changes in the frequency and amplitude produced by the interaction perturbation are calculated. Here, the frequency is increased and the amplitude is decreased.

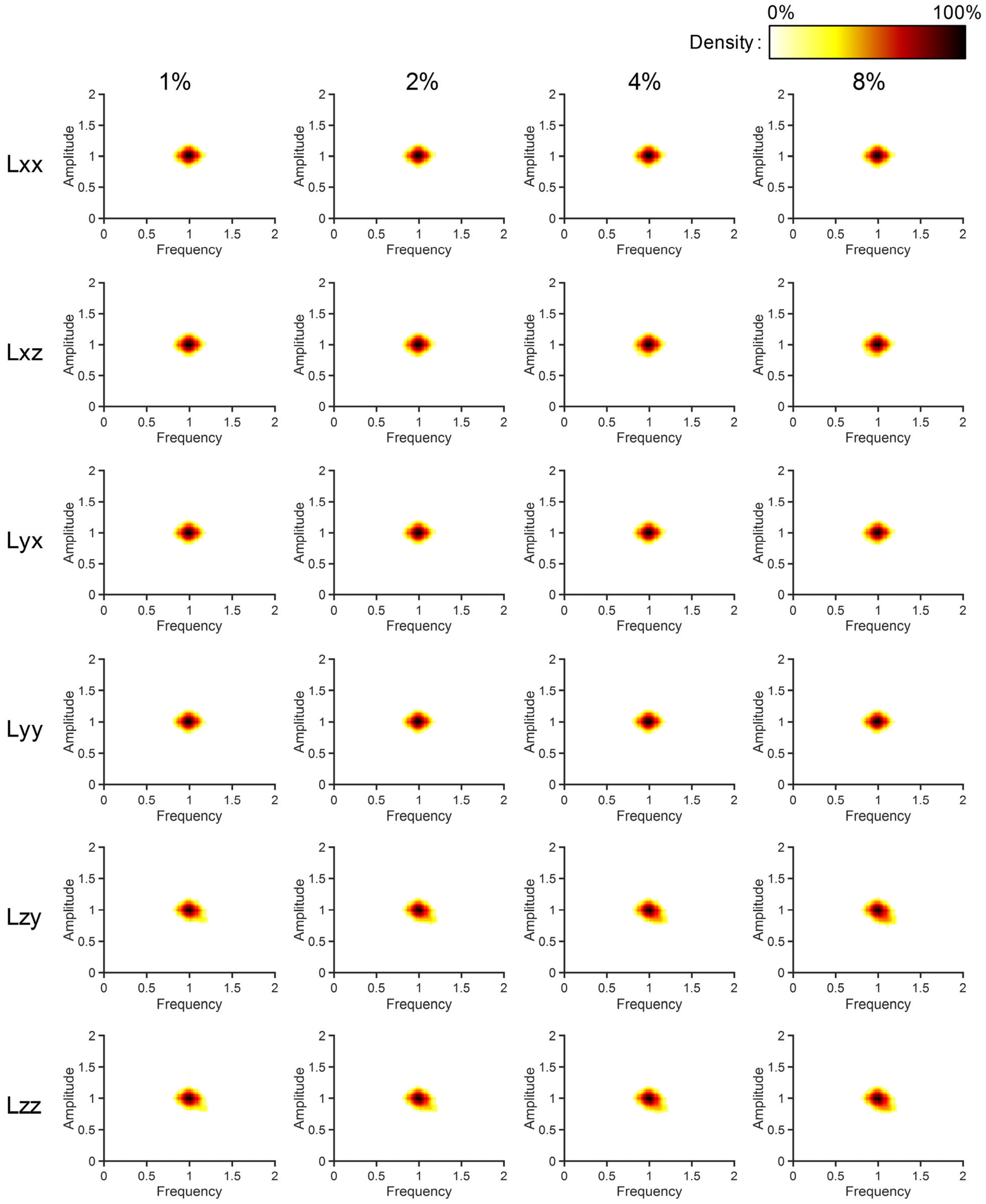

Additional Figure A2. Density plots of the simple NFO. Both frequency and amplitude are hardly modulated regardless of the type of perturbed link.

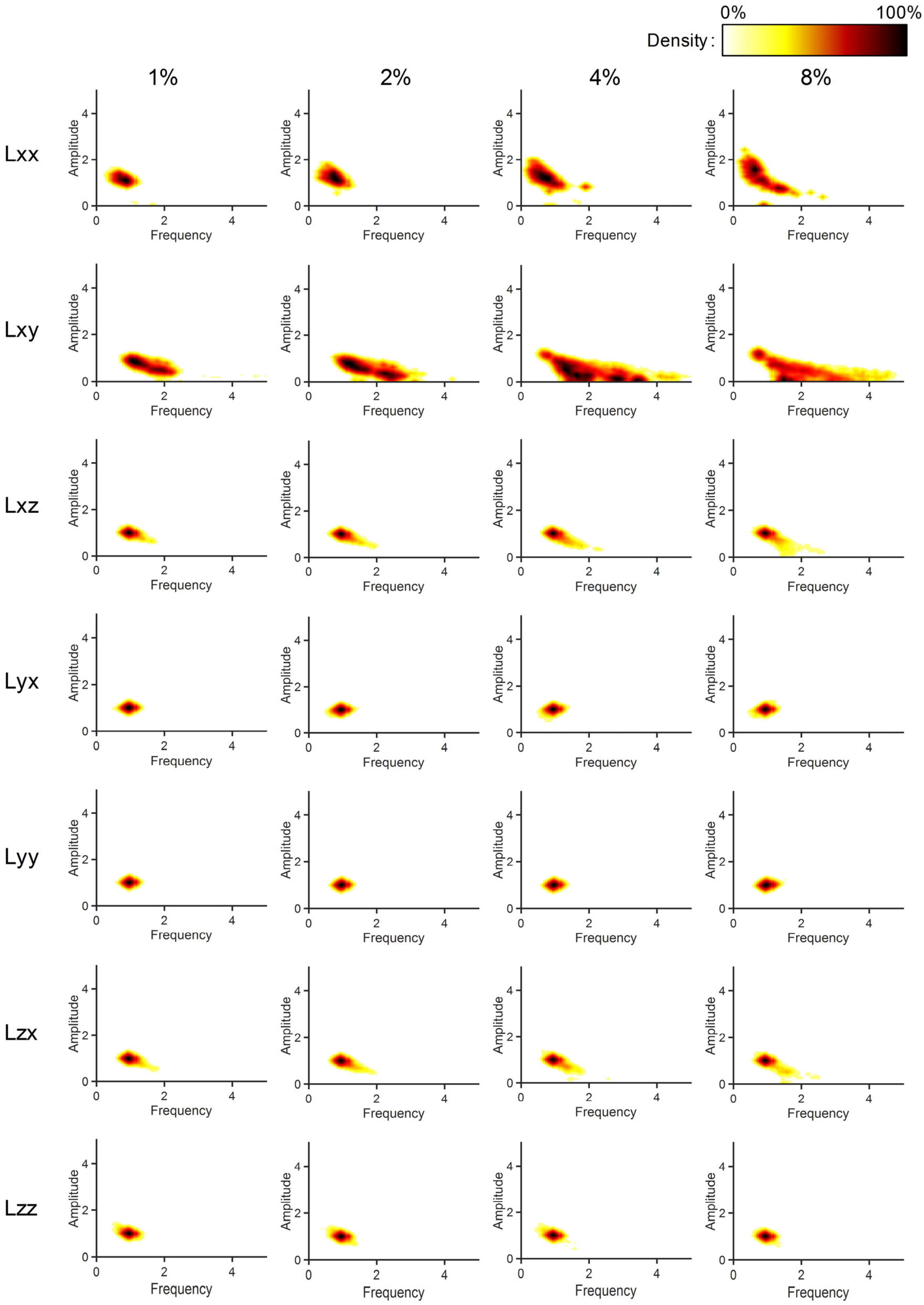

Additional Figure A3. Density plots of the activator-amplified NFO. Changes in frequency were larger than changes in amplitude. Perturbation of Lxx and Lxy mainly resulted in changes in frequency.

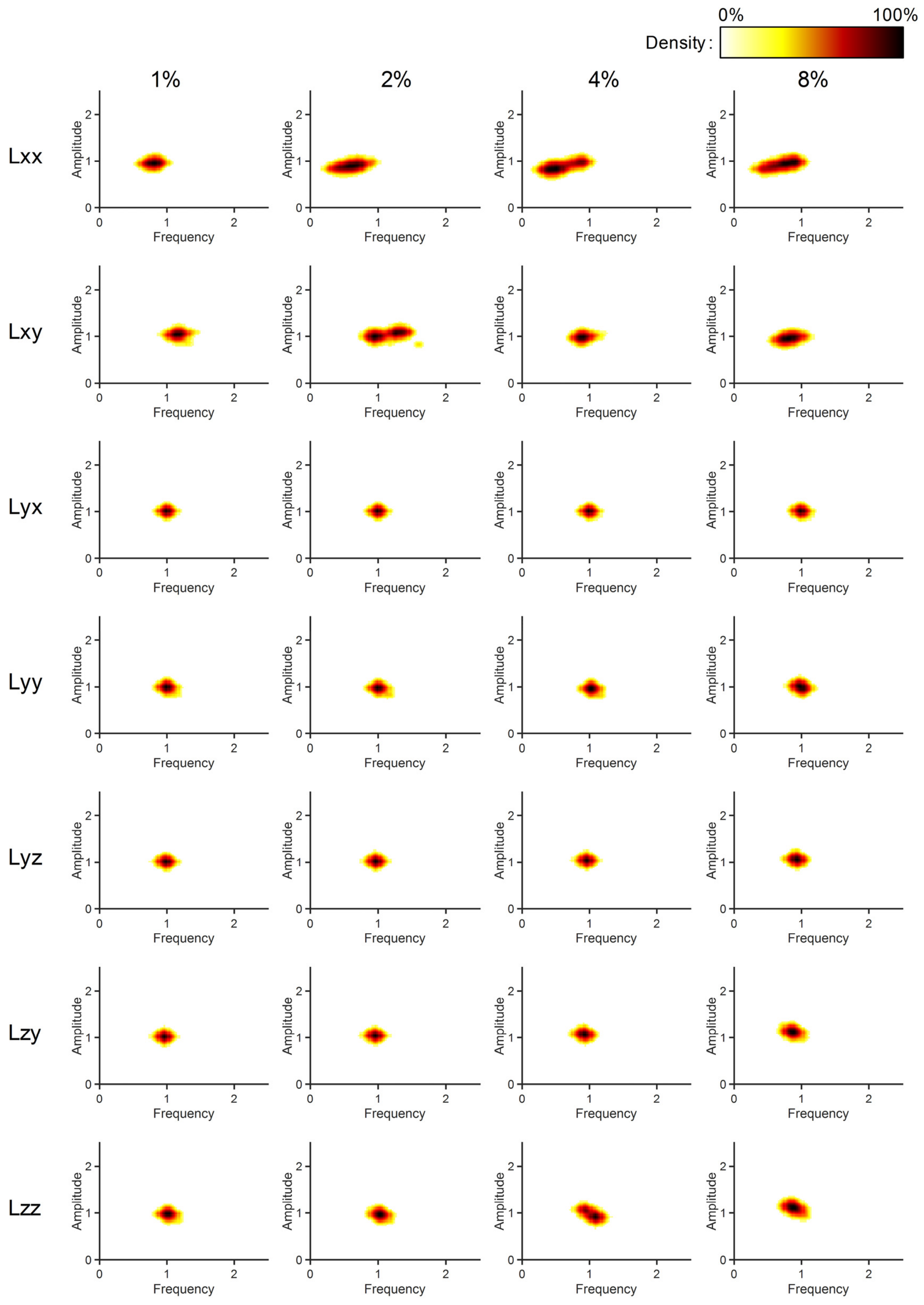

Additional Figure A4. Density plots of the inhibitor-amplified NFO. Changes in frequency were larger than changes in amplitude. Perturbation of Lxx and Lxy mainly resulted in changes in frequency.

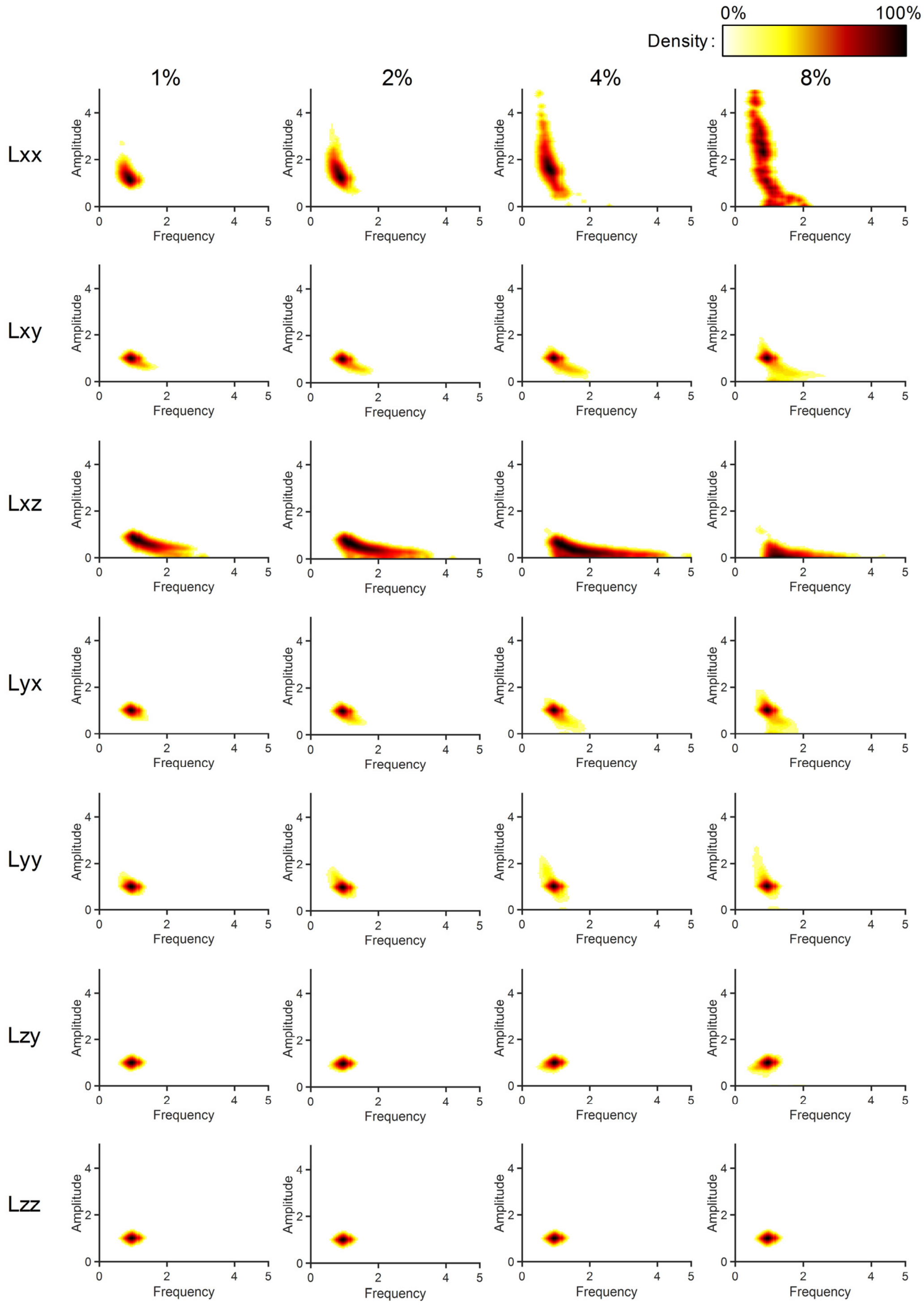

Additional Figure A5. Density plots of the type 1 incoherently-amplified NFO. Changes in amplitude were larger than changes in frequency. Perturbation of Lxx and Lxz mainly resulted in changes in amplitude.

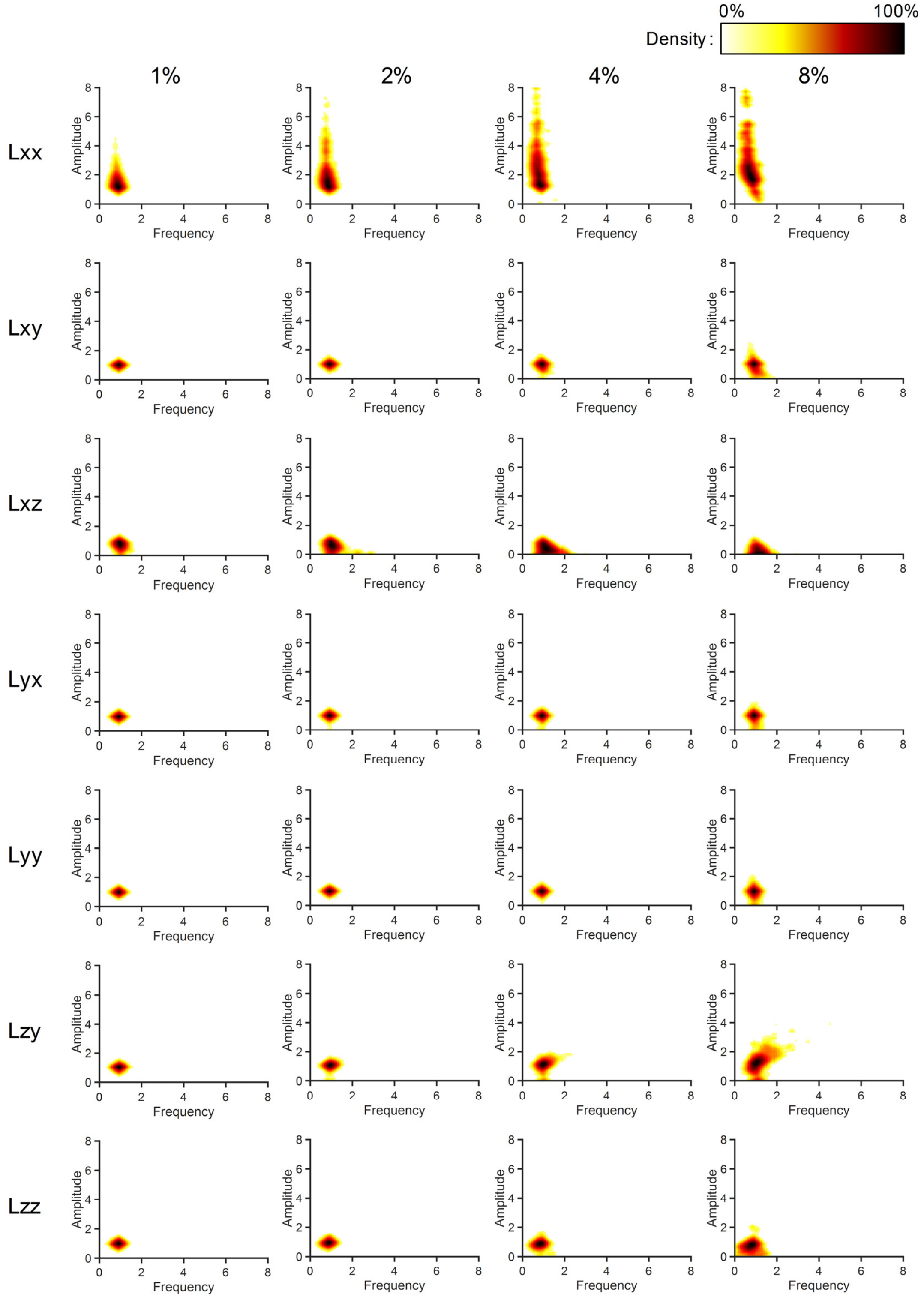

Additional Figure A6. Density plots of the type 2 incoherently-amplified NFO. Changes in amplitude were larger than changes in frequency. Perturbation of Lxx and Lxz mainly resulted in changes in amplitude.

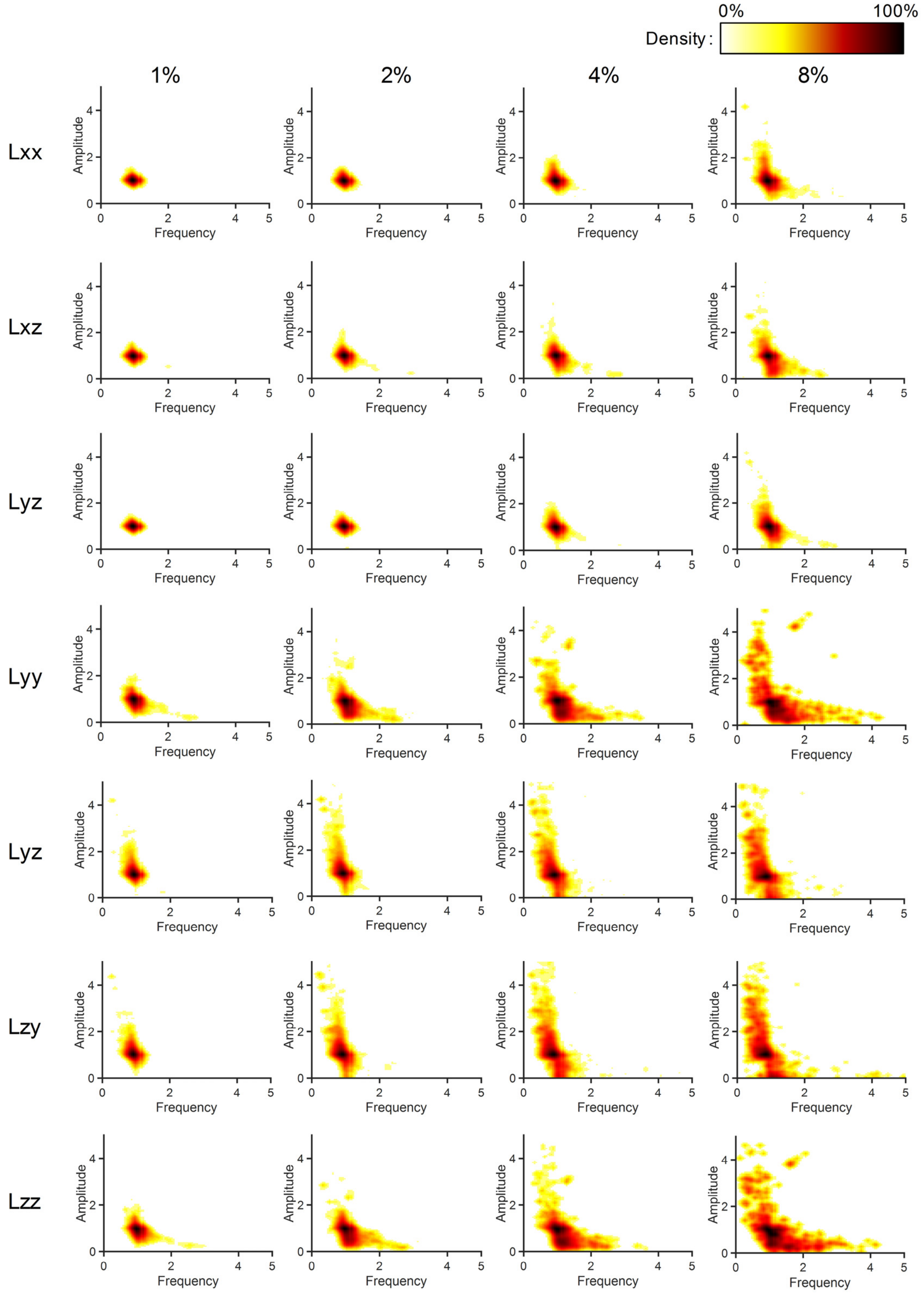

Additional Figure A7. Density plots of the type 3 incoherently-amplified NFO. Changes in amplitude were larger than changes in frequency. Perturbation of Lyy, Lyz, Lzy and Lzz mainly resulted in changes in amplitude.

II. Additional Tables

Additional Table A1. Regulatory patterns of the frequency and amplitude according to the types of interactions.

Network structure of oscillators	Strength of perturbation	type of interaction	Pattern R[a]	Pattern F[b]	Pattern A[c]
Simple NFO	1% weakening	Lxx	98.88%	0.01%	1.11%
		Lxz	95.29%	0.82%	3.89%
		Lyx	98.78%	0.05%	1.17%
		Lyy	99.58%	0%	0.42%
		Lzy	79.88%	1.1%	19.02%
		Lzz	82.78%	1.14%	16.08%
	2% weakening	Lxx	98.02%	0.03%	1.95%
		Lxz	92.98%	1.04%	5.98%
		Lyx	97.52%	0.13%	2.35%
		Lyy	99.38%	0%	0.62%
		Lzy	72.92%	0.82%	26.26%
		Lzz	76.82%	0.8%	22.38%
	4% weakening	Lxx	96.79%	0.1%	3.11%
		Lxz	90.84%	1.28%	7.88%
		Lyx	95.24%	0.43%	4.33%
		Lyy	98.88%	0.03%	1.09%
		Lzy	66.28%	0.65%	33.08%
		Lzz	70.85%	0.63%	28.52%

	8% weakening	Lxx	94.78%	0.26%	4.97%
		Lxz	88.53%	1.62%	9.85%
		Lyx	91.59%	1.03%	7.39%
		Lyy	98.02%	0.12%	1.86%
		Lzy	61.3%	0.54%	38.16%
		Lzz	65.49%	0.53%	33.98%
Activator-amplified NFO	1% weakening	Lxx	2.85%	74.02%	23.13%
		Lxy	0.52%	90.06%	9.42%
		Lxz	71.8%	23.64%	4.55%
		Lyx	91.59%	7.49%	0.91%
		Lyy	97.95%	1.85%	0.2%
		Lzx	71.92%	23.43%	4.65%
		Lzz	76.71%	17.86%	5.43%
	2% weakening	Lxx	2.05%	72.59%	25.36%
		Lxy	0.53%	89.78%	9.68%
		Lxz	68.85%	26.05%	5.1%
		Lyx	88.72%	9.8%	1.48%
		Lyy	96.4%	3.23%	0.38%
		Lzx	68.87%	26%	5.12%
		Lzz	76.19%	18.17%	5.64%
	4% weakening	Lxx	1.81%	71.77%	26.42%
		Lxy	0.37%	90%	9.63%
		Lxz	65.44%	28.76%	5.81%
		Lyx	85.11%	12.84%	2.05%

		Lyy	93.97%	5.44%	0.58%
		Lzx	65.52%	28.75%	5.72%
		Lzz	75.1%	19.39%	5.51%
	8%weakening	Lxx	1.79%	70.99%	27.22%
		Lxy	0.32%	90.16%	9.51%
		Lxz	61.06%	32.5%	6.43%
		Lyx	81.36%	15.02%	3.62%
		Lyy	89.71%	9.36%	0.93%
		Lzx	61.24%	32.33%	6.43%
		Lzz	72.26%	22.15%	5.6%
Inhibitor-amplified NFO	1% weakening	Lxx	0.05%	99.93%	0.02%
		Lxy	0.06%	99.89%	0.06%
		Lyx	99.04%	0.38%	0.58%
		Lyy	48.58%	7.7%	43.71%
		Lyz	55.61%	8.9%	35.5%
		Lzy	0.48%	61.64%	37.88%
		Lzz	1.08%	67.64%	31.28%
	2% weakening	Lxx	0.04%	99.87%	0.09%
		Lxy	0.78%	99.17%	0.05%
		Lyx	98.08%	0.43%	1.49%
		Lyy	30.13%	14.02%	55.85%
		Lyz	28.49%	22.05%	49.46%
		Lzy	1.12%	62.66%	36.22%
		Lzz	1.91%	70.42%	27.66%

	4% weakening	Lxx	0.02%	99.86%	0.12%
		Lxy	1.15%	98.81%	0.04%
		Lyx	96.77%	0.7%	2.53%
		Lyy	27.55%	14.03%	58.42%
		Lyz	19.8%	30.38%	49.82%
		Lzy	1.23%	63.85%	34.92%
		Lzz	1.96%	71.44%	26.59%
	8% weakening	Lxx	0.01%	99.84%	0.15%
		Lxy	1.21%	98.76%	0.03%
		Lyx	93.7%	1.9%	4.4%
		Lyy	27.54%	14.05%	58.41%
		Lyz	15.46%	38.17%	46.38%
		Lzy	1.08%	64.68%	34.24%
		Lzz	1.95%	70.71%	27.34%
Type 1 incoherently-amplified NFO	1% weakening	Lxx	0.46%	0%	99.54%
		Lxy	64.27%	0.56%	35.17%
		Lxz	1.02%	5.99%	92.99%
		Lyx	71.22%	0.1%	28.68%
		Lyy	71.12%	0.15%	28.73%
		Lzy	94.44%	3.01%	2.56%
		Lzz	100%	0%	0%
	2% weakening	Lxx	0.98%	0%	99.02%
		Lxy	56.76%	1.08%	42.16%
		Lxz	1.04%	15.02%	83.94%

		Lyx	60.39%	0.15%	39.46%
		Lyy	59.32%	0.25%	40.43%
		Lzy	87.01%	5.82%	7.17%
		Lzz	98.85%	0.05%	1.1%
	4% weakening	Lxx	1.25%	0.31%	98.43%
		Lxy	46.95%	2.65%	50.4%
		Lxz	0.92%	20.02%	79.05%
		Lyx	47.24%	0.52%	52.24%
		Lyy	47.53%	0.16%	52.3%
		Lzy	73.66%	9.54%	16.8%
		Lzz	96.95%	0.85%	2.2%
	8% weakening	Lxx	0%	0%	100%
		Lxy	37.69%	3.56%	58.75%
		Lxz	1.66%	4.05%	94.29%
		Lyx	38.11%	0.58%	61.31%
		Lyy	37.28%	0.19%	62.53%
		Lzy	60.29%	12.82%	26.89%
		Lzz	89.23%	5.51%	5.26%
Type 2 incoherently-amplified NFO	1% weakening	Lxx	0%	0%	100%
		Lxy	73.03%	0.1%	26.87%
		Lxz	0.52%	0%	99.48%
		Lyx	90.85%	0%	9.15%
		Lyy	91.64%	0.05%	8.31%
		Lzy	6.46%	17.9%	75.64%

		Lzz	6.15%	17.44%	76.41%
	2% weakening	Lxx	0.08%	0%	99.92%
		Lxy	59.87%	0.05%	40.08%
		Lxz	0.33%	1.32%	98.35%
		Lyx	78.75%	0.1%	21.15%
		Lyy	73.47%	0.1%	26.43%
		Lzy	1.4%	21.42%	77.18%
		Lzz	0.51%	16.92%	82.56%
	4% weakening	Lxx	0%	0%	100%
		Lxy	48.66%	0.11%	51.23%
		Lxz	0.14%	1.64%	98.22%
		Lyx	55.14%	0.1%	44.76%
		Lyy	47.14%	0.1%	52.75%
		Lzy	1.18%	20%	78.82%
		Lzz	0%	15.11%	84.89%
	8% weakening	Lxx	0%	0%	100%
		Lxy	35.47%	0.05%	64.47%
		Lxz	0.42%	0%	99.58%
		Lyx	34.84%	0.05%	65.1%
		Lyy	25.68%	0.11%	74.22%
		Lzy	0.86%	16.28%	82.87%
		Lzz	0%	10.76%	89.24%
Type 3 incoherently-amplified NFO	1% weakening	Lxx	30.24%	13.4%	56.36%
		Lxz	32.61%	14.12%	53.28%

		Lyx	30.77%	15%	54.23%
		Lyy	8.14%	29.3%	62.56%
		Lyz	10.25%	27.58%	62.17%
		Lzy	4.02%	31.84%	64.14%
		Lzz	4.73%	30.65%	64.62%
	2% weakening	Lxx	19.82%	19.74%	60.44%
		Lxz	23.86%	18.82%	57.32%
		Lyx	18.98%	20.55%	60.47%
		Lyy	4.37%	31.91%	63.73%
		Lyz	5.73%	31.06%	63.21%
		Lzy	1.77%	32.26%	65.97%
		Lzz	2.41%	32.28%	65.31%
	4% weakening	Lxx	12.11%	25.97%	61.92%
		Lxz	15.72%	23.99%	60.29%
		Lyx	10.3%	26.03%	63.67%
		Lyy	2.42%	35.22%	62.36%
		Lyz	2.93%	32.61%	64.46%
		Lzy	0.83%	33.12%	66.05%
		Lzz	1.36%	33.43%	65.21%
	8% weakening	Lxx	6.78%	30.74%	62.48%
		Lxz	9.13%	28.09%	62.77%
		Lyx	6.63%	28.25%	65.13%
		Lyy	1.36%	37.41%	61.22%
		Lyz	2.09%	35.18%	62.73%

		Lzy	0.78%	34.98%	64.24%
		Lzz	1.37%	36.32%	62.31%

[a]Both the frequency and amplitude changed by less than 1%.

[b]The frequency or amplitude changed by more than 1%, and the changes in the frequency are greater than the changes in the amplitude.

[c]The frequency or amplitude changed by more than 1%, and the changes in the amplitude are greater than the frequency.

Additional Table A2. Relative mean differences between 1st-order ODE and 2nd-order ODE in the results of the simulations during ten periods of oscillations. Each relative mean difference was calculated by dividing the absolute difference by the maximum value of oscillation of each oscillator. For six representative 3-node oscillator models (*), each relative mean difference was calculated for each parameter set and represented by mean ± standard deviation.

**Oscillator**	**The relative mean difference**
Simple NFO*	0.00039 ± 0.0021
Activator-amplified NFO*	0.000023 ± 0.00013
Inhibitor-amplified NFO*	0.0012 ± 0.0032
Type 1 incoherently-amplified NFO*	0.0093 ± 0.011
Type 2 incoherently-amplified NFO*	0.0000059 ± 0.000033
Type 3 incoherently-amplified NFO*	0.042 ± 0.11
Circadian rhythm model by Leloup, et al.	0.00064
Circadian rhythm model by Goldbeter, et al.	0.0170
Repressilator	0.000029
Sinus node model by Yanagihara, et al.	0.00023

Hodgkin-Huxley axon model	0.00087
Cell cycle model by Ferrell, et al.	0.000030
cAMP model by Martiel, et al.	0.0000060
Glycolysis model by Selkov model	0.000065
Glycolysis model by Higgins model	0.000020

Additional Table A3. Parameter sets for the 3-node biochemical oscillator models for mathematically controlled comparisons.

Model	Parameter set
Simple NFO	$k_{sx} = 0.1, S = 2, k_{dx} = 0.1, p = 4, k_1 = 0.2, k_2 = 0.1, K_m = 0.01, k_3 = 0.05$
Activator-amplified NFO variant	$k_{sx} = 0.012, S = 2, k_{dx} = 0.1, p = 4, k_1 = 0.2, k_2 = 0.1, K_m = 0.01, k_3 = 0.05,$ $k_{amp} = 0.188, q = 2$
Inhibitor-amplified NFO variant	$k_{sx} = 0.1, S = 2, k_{dx} = 0.1, p = 4, k_1 = 0.047, k_2 = 0.1, K_m = 0.01, k_3 = 0.05,$ $k_{amp} = 0.07, q = 2$
Type 1 incoherently-amplified NFO variant	$k_{sx} = 0.027, S = 2, k_{dx} = 0.1, p = 4, k_1 = 0.2, k_2 = 0.1, K_m = 0.01, k_3 = 0.05,$ $k_{inc} = 0.052, q = 2$

Additional Table A4. Percentage of parameter sets for the 3-node biochemical oscillator models that did not sustain limit-cycle oscillation after interaction perturbation from total parameter sets.

Oscillator	Perturbation Strength: 1%	Perturbation Strength: 2%	Perturbation Strength: 4%	Perturbation Strength: 8%
Type 1 simple NFO	0.31%	0.66%	2.25%	6.18%
Activator-amplified NFO	12.08%	15.38%	22.32%	34.06%

Inhibitor-amplified NFO	5.67%	13.92%	39.77%	59.55%
Type 1 incoherently-amplified NFO*	11.01%	14.62%	20.75%	33.62%
Type 2 incoherently-amplified NFO*	16.71%	23.15%	28.44%	34.58%
Type 3 incoherently-amplified NFO*	0.73%	2.34%	6.78%	21.91%

III. Additional Equations

Additional Equation A1. ODEs of the 3-node biochemical oscillators

State variables: x, y, z

Time derivatives of state variables: $\frac{dx}{dt}, \frac{dy}{dt}, \frac{dz}{dt}$

(1) Simple NFO

$$\frac{dx}{dt} = \frac{S \times k_{sx}}{z^p + 1} - k_{dx} \times x$$
$$\frac{dy}{dt} = k_1 \times x - \frac{k_2 \times y}{K_m + y}$$
$$\frac{dz}{dt} = k_3 \times (y - z)$$

(2) Activator-amplified NFO

$$\frac{dx}{dt} = k_{sx\_basal} + k_{sx} \times z - (k_{dx\_basal} + k_{dx} \times y) \times x$$
$$\frac{dy}{dt} = \frac{k_{sy} \times x^p}{1 + x^p} - k_{dy} \times y$$
$$\frac{dz}{dt} = \frac{k_{sz} \times x^q}{1 + x^q} - k_{dz} \times z$$

(3) Inhibitor-amplified NFO

$$\frac{dx}{dt} = k_{sx} - (k_{dx\_basal} + k_{dx} \times y^p) \times x$$
$$\frac{dy}{dt} = k_{sy\_basal} + k_{sy} \times x - (k_{dy\_basal} + k_{dy} \times (ZT - z)) \times y$$
$$\frac{dz}{dt} = \frac{k_1 \times y \times (ZT - z)}{K_{m1} + (ZT - z)} - \frac{k_2 \times z}{K_{m2} + z}$$

(4) Type 1 incoherently-amplified NFO

$$\frac{dx}{dt} = \frac{k_{sx} \times y^q}{1 + y^q} - k_{dx} \times z \times x$$
$$\frac{dy}{dt} = k_{sy\_basal} + k_{sy} \times x - k_{dy} \times y$$
$$\frac{dz}{dt} = \frac{k_{sz} \times y^p}{1 + y^p} - k_{dz} \times z$$

(5) Type 2 incoherently-amplified NFO

$$\frac{dx}{dt} = k_{sx\_basal} + k_{xz} \times x \times z + \frac{k_{sx} \times y^q}{y^q + 1} - k_{dx} \times x$$
$$\frac{dy}{dt} = k_3 \times (x - y)$$
$$\frac{dz}{dt} = k_{sz} - k_{dz} \times \frac{y^q}{y^q + 1} \times z$$

(6) Type 3 incoherently-amplified NFO

$$\frac{dx}{dt} = \frac{k_{sx} \times (ZT - z)}{(ZT - z) + K_{mx}} - k_{dx} \times x$$
$$\frac{dy}{dt} = k_{sy} \times x - (k_{dy\_basal} + k_{dy} \times (ZT - z)) \times y$$
$$\frac{dz}{dt} = \frac{k_1 \times y \times (ZT - z)}{K_{m1} + (ZT - z)} - \frac{k_2 \times z}{K_{m2} + z}$$

(7) Activator-amplified NFO variant

$$\frac{dx}{dt} = \frac{S \times k_{sx}}{z^p + 1} + k_{amp} \times \frac{x^q}{1 + x^q} - k_{dx} \times x$$
$$\frac{dy}{dt} = k_1 \times x - \frac{k_2 \times y}{K_m + y}$$
$$\frac{dz}{dt} = k_3 \times (y - z)$$

(8) Inhibitor-amplified NFO variant

$$\frac{dx}{dt} = \frac{S \times k_{sx}}{z^p + 1} - k_{dx} \times x$$
$$\frac{dy}{dt} = k_1 \times x + k_{amp} \times \frac{y^q}{1 + y^q} - \frac{k_2 \times y}{K_m + y}$$
$$\frac{dz}{dt} = k_3 \times (y - z)$$

(9) Type 1 incoherently-amplified NFO variant

$$\frac{dx}{dt} = \frac{S \times k_{sx}}{z^p + 1} + k_{inc} \times \frac{y^q}{1 + y^q} - k_{dx} \times x$$
$$\frac{dy}{dt} = k_1 \times x - \frac{k_2 \times y}{K_m + y}$$
$$\frac{dz}{dt} = k_3 \times (y - z)$$

Additional Equation A2. ODEs and parameters for naturally occurring biochemical oscillator models

(1) Circadian rhythm model by Leloup *et al.*

State variables: $M_P, M_C, M_B, P_C, C_C, P_{CP}, C_{CP}, PC_C, PC_N, PC_{CP}, PC_{NP}, B_C, B_{CP}, B_N, B_{NP}, I_N$

Time derivatives of state variables: $\frac{dM_P}{dt}, \frac{dM_C}{dt}, \frac{dM_B}{dt}, \frac{dP_C}{dt}, \frac{dC_C}{dt}, \frac{dP_{CP}}{dt}, \frac{dC_{CP}}{dt},$

$\frac{dPC_C}{dt}, \frac{dPC_N}{dt}, \frac{dPC_{CP}}{dt}, \frac{dPC_{NP}}{dt}, \frac{dB_C}{dt}, \frac{dB_{CP}}{dt}, \frac{dB_N}{dt}, \frac{dB_{NP}}{dt}, \frac{dI_N}{dt}$

$$\frac{dM_P}{dt} = \frac{v_{sp} \times B_N{}^n}{K_{AP}{}^n + B_N{}^n} - \frac{v_{mP} \times M_P}{K_{mP} + M_P} - k_{dmp} \times M_P$$

$$\frac{dM_C}{dt} = \frac{v_{sC} \times B_N{}^n}{K_{AC}{}^n + B_N{}^n} - \frac{v_{mC} \times M_C}{K_{mC} + M_C} - k_{dmc} \times M_C$$

$$\frac{dM_B}{dt} = \frac{v_{sB} \times K_{IB}{}^m}{K_{IB}{}^m + B_N{}^m} - \frac{v_{mB} \times M_B}{K_{mB} + M_B} - k_{dmb} \times M_B$$

$$\frac{dP_C}{dt} = k_{sP} \times M_P - \frac{V_{1P} \times P_C}{K_p + P_C} + \frac{V_{2P} \times P_{CP}}{K_{dp} + P_{CP}} + k_4 \times PC_C - k_3 \times P_C \times C_C - k_{dn} \times P_C$$

$$\frac{dC_C}{dt} = k_{sC} \times M_C - \frac{V_{1C} \times C_C}{K_p + C_C} + \frac{V_{2C} \times C_{CP}}{K_{dp} + C_{CP}} + k_4 \times PC_C - k_3 \times P_C \times C_C - k_{dnc} \times C_C$$

$$\frac{dP_{CP}}{dt} = \frac{V_{1P} \times P_C}{K_p + P_C} - \frac{V_{2P} \times P_{CP}}{K_{dp} + P_{CP}} - \frac{v_{dPC} \times P_{CP}}{K_d + P_{CP}} - k_{dn} \times P_{CP}$$

$$\frac{dC_{CP}}{dt} = \frac{V_{1C} \times C_C}{K_p + C_C} - \frac{V_{2C} \times C_{CP}}{K_{dp} + C_{CP}} - \frac{v_{dCC} \times C_{CP}}{K_d + C_{CP}} - k_{dn} \times C_{CP}$$

$$\frac{dPC_C}{dt} = -\frac{V_{1PC} \times PC_C}{K_p + PC_C} + \frac{V_{2PC} \times PC_{CP}}{K_{dp} + PC_{CP}} - k_4 \times PC_C + k_3 \times P_C \times C_C + k_2 \times PC_N - k_1 \times PC_C - k_{dn} \times PC_C$$

$$\frac{dPC_N}{dt} = -\frac{V_{3PC} \times PC_N}{K_p + PC_N} + \frac{V_{4PC} \times PC_{NP}}{K_{dp} + PC_{NP}} - k_2 \times PC_N + k_1 \times PC_C - k_7 \times B_N \times PC_N + k_8 \times I_N - k_{dn} \times PC_N$$

$$\frac{dPC_{CP}}{dt} = \frac{V_{1PC} \times PC_C}{K_p + PC_C} - \frac{V_{2PC} \times PC_{CP}}{K_{dp} + PC_{CP}} - \frac{v_{dPCC} \times PC_{CP}}{K_d + PC_{CP}} - k_{dn} \times PC_{CP}$$

$$\frac{dPC_{NP}}{dt} = \frac{V_{3PC} \times PC_N}{K_p + PC_N} - \frac{V_{4PC} \times PC_{NP}}{K_{dp} + PC_{NP}} - \frac{v_{dPCN} \times PC_{NP}}{K_d + PC_{NP}} - k_{dn} \times PC_{NP}$$

$$\frac{dB_C}{dt} = k_{sB} \times M_B - \frac{V_{1B} \times B_C}{K_p + B_C} + \frac{V_{2B} \times B_{CP}}{K_{dp} + B_{CP}} - k_5 \times B_C + k_6 \times B_N - k_{dn} \times B_C$$

$$\frac{dB_{CP}}{dt} = \frac{V_{1B} \times B_C}{K_p + B_C} - \frac{V_{2B} \times B_{CP}}{K_{dp} + B_{CP}} - \frac{v_{dBC} \times B_{CP}}{K_d + B_{CP}} - k_{dn} \times B_{CP}$$

$$\frac{dB_N}{dt} = \frac{V_{3B} \times B_N}{K_p + B_N} - \frac{V_{4B} \times B_{NP}}{K_{dp} + B_{NP}} + k_5 \times B_C - k_6 \times B_N - k_7 \times B_N \times PC_N + k_8 \times I_N - k_{dn} \times B_N$$

$$\frac{dB_{NP}}{dt} = \frac{V_{3B} \times B_N}{K_p + B_N} - \frac{V_{4B} \times B_{NP}}{K_{dp} + B_{NP}} - \frac{v_{dBN} \times B_{NP}}{K_d + B_{NP}} - k_{dn} \times B_{NP}$$

$$\frac{dI_N}{dt} = -k_8 \times I_N + k_7 \times B_N \times PC_N - \frac{v_{dIN} \times I_N}{K_d + I_N} - k_{dn} \times I_N$$

$n = 4, m = 4, k_{stot} = 1, k_{sP} = 0.5 \times k_{stot}, k_{sC} = k_{stot}, k_{sB} = k_{stot}$

$v_{stot} = 1, v_{sP} = v_{stot}, v_{sC} = 0.8 \times v_{stot}, v_{sB} = 0.7 \times v_{stot}, K_{AP} = 0.7, K_{AC} = 1, K_{IB} = 0.8$

$v_{mP} = 1.1, v_{mC} = 1, v_{mB} = 0.2, K_{mP} = 0.3, K_{mC} = 0.4, K_{mB} = 0.4, k_{dmp} = 0.01, k_{dmc} = 0.01, k_{dmb} = 0.01$

$V_{phos} = 0.6, V_{1B} = 1, V_{1C} = 0.6, V_{1P} = V_{phos}, V_{1PC} = V_{phos}, V_{2B} = 0.1, V_{2C} = 0.1, V_{2P} = 0.3, V_{2PC} = 0.1, V_{3B} = 1, V_{3PC} = V_{phos}$

$V_{4B} = 0.2, V_{4PC} = 0.1, k_1 = 0.8, k_2 = 0.2, k_3 = 0.8, k_4 = 0.2, k_5 = 0.4, k_6 = 0.2, k_7 = 0.5, k_8 = 0.1, k_{dn} = 0.01$

$k_{dnc} = 0.01, K_d = 0.3, K_p = 0.1, K_{dp} = 0.3, v_{dBC} = 1, v_{dBN} = 0.5, v_{dCC} = 0.7, v_{dIN} = 0.8, v_{dPC} = 0.7, v_{dPCC} = 1, v_{dPCN} = 1$

(2) Circadian rhythm model by Goldbeter.

State variables: $x1, x2, x3, x4$

Time derivatives of state variables: $\frac{dx1}{dt}, \frac{dx2}{dt}, \frac{dx3}{dt}, \frac{dx4}{dt}$

$$\frac{dx1}{dt} = \frac{1}{Cytoplasm} \times (rM - rmRNAd)$$

$$\frac{dx2}{dt} = \frac{1}{Cytoplasm} \times (rTL - rP01 + rP10)$$

$$\frac{dx3}{dt} = \frac{1}{Cytoplasm} \times (rP01 - rP10 - rP12 + rP21)$$

$$\frac{dx4}{dt} = \frac{1}{Cytoplasm} \times (rP12 - rP21 - rP2n + rPn2 - rVd)$$

$$\frac{dx5}{dt} = \frac{1}{r4} \times (rP2n - rPn2)$$

$default = 1.0 \times 10^{-15}, Cytoplasm = 1.0 \times 10^{-15}, r4 = 1.0 \times 10^{-15}, rM_{Vs} = 0.76, rM_{KI} = 1.0, rM_n = 4.0$

$$rM = default \times rM_{Vs} \times \frac{rM_{KI}^{rM_n}}{rM_{KI}^{rM_n} + x5^{rM_n}}$$

$$rP01_{V1} = 3.2, rP01_{K1} = 2.0, rP01 = Cytoplasm \times \frac{rP01_{V1} \times x2}{rP01_{K1} + x2}$$

$$rP10_{V2} = 1.58, rP10_{K2} = 2.0, rP10 = Cytoplasm \times \frac{rP10_{V2} \times x3}{rP10_{K2} + x3}$$

$$rP12_{V3} = 5.0, rP12_{K3} = 2.0, rP12 = Cytoplasm \times \frac{rP12_{V3} \times x3}{rP12_{K3} + x3}$$

$$rP21_{V4} = 2.5, rP21_{K4} = 2.0, rP21 = Cytoplasm \times \frac{rP21_{V4} + x4}{rP21_{K4} + x4}$$

(3) Repressilator by Elowitz and Leibler (modified version).

State variables: x, y, z

Time derivatives of state variables: $\frac{dx}{dt}, \frac{dy}{dt}, \frac{dz}{dt}$

$$\frac{dx}{dt} = -\frac{k_d \times x}{1+x} + \frac{k_s}{1+z^n} + \frac{k_{amp} \times x^2}{1+x^2}$$
$$\frac{dy}{dt} = -\frac{k_d \times y}{1+y} + \frac{k_s}{1+x^n}$$
$$\frac{dz}{dt} = -\frac{k_d \times z}{1+z} + \frac{k_s}{1+y^n}$$

$n = 3, k_s = 0.07, k_d = 0.1, k_{amp} = 0.07$

(4) Sinus node model by Yanagihara *et al.*

State variables: V, m, h, n, d, f, q

Time derivatives of state variables: $\frac{dV}{dt}, \frac{dm}{dt}, \frac{dh}{dt}, \frac{dn}{dt}, \frac{dd}{dt}, \frac{df}{dt}, \frac{dq}{dt}$

$$\frac{dV}{dt} = \frac{-(i_{Na} + i_K + i_{Leak} + i_s + i_{h)}}{Cm}$$
$$\frac{dm}{dt} = \alpha_m \times (1-m) - \beta_m \times m$$

$$\frac{dh}{dt} = \alpha_h \times (1-h) - \beta_h \times h$$
$$\frac{dn}{dt} = \alpha_n \times (1-n) - \beta_n \times n$$

$$\frac{dd}{dt} = \alpha_d \times (1-d) - \beta_d \times d$$
$$\frac{df}{dt} = \alpha_f \times (1-f) - \beta_f \times f$$
$$\frac{dq}{dt} = \alpha_q \times (1-q) - \beta_q \times q$$

$$Cm = 1$$
$$\alpha_f = \frac{-0.000355 \times (V+20)}{-\exp(\frac{V+20}{5.633}) + 1}, \beta_f = \frac{0.000944 \times (V+60)}{1+\exp(-\frac{V+29.5}{4.16})}$$
$$\alpha_n = \frac{0.009}{1+\exp(-\frac{V+3.8}{9.71})} + 0.0006, \beta_n = \frac{-0.000225 \times (V+40)}{1-\exp(\frac{V+40}{13.3})}$$

$$\alpha_q = \frac{0.00034 \times (V+100)}{\exp(\frac{V+100}{4.4} - 1)} - 0.0000495, \beta_q = \frac{0.0005 \times (V+40)}{1-\exp(-\frac{V+40}{6})} + 0.0000845$$
$$\alpha_m = \frac{V+37}{-\exp(-\frac{V+37}{10}) + 1}, \beta_m = 40 \times \exp(-\frac{V+62}{17.8})$$

$$\alpha_d = \frac{0.0145 \times (V+35)}{1-\exp(-\frac{V+35}{2.5})} + \frac{0.03125 \times V}{1-\exp(-\frac{V}{4.8})}, \beta_d = -\frac{0.00421 \times (V-5)}{-\exp(\frac{V-5}{2.5}) + 1}$$
$$\alpha_h = 0.001209 \times \exp(\frac{V+20}{-6.534}), \beta_h = \frac{1}{1+\exp(-\frac{V+30}{10})}$$

$$i_s = 12.5 \times (0.95 \times d + 0.05) \times (0.95 \times f + 0.05) \times (\exp(\frac{V-10}{15}) - 1)$$
$$i_{Na} = 0.5 \times m^3 \times h \times (V-30)$$
$$i_{Leak} = 0.8 \times (1-\exp(-\frac{V+60}{20}))$$
$$i_h = 0.4 \times q \times (V+45)$$
$$i_K = \frac{0.7 \times n \times (\exp(0.0277 \times (V+90)) - 1)}{\exp(0.0277 \times (V+40))}$$

(5) Neuronal model by Hodgkin and Huxley

State variables: V, m, h, n

Time derivatives of state variables: $\frac{dV}{dt}, \frac{dm}{dt}, \frac{dh}{dt}, \frac{dn}{dt}$

$$\frac{dV}{dt} = I - g_{Na} \times m^3 \times h \times (V - v_{Na}) - g_K \times n^4 \times (V - v_K) - g_L \times (V - v_L)$$

$$\frac{dm}{dt} = (1 - m) \times a_M - m \times b_M$$

$$\frac{dh}{dt} = (1 - h) \times a_H - h \times b_H$$

$$\frac{dn}{dt} = (1 - n) \times a_N - n \times b_N$$

$$C = 1, g_{Na} = 120, g_K = 36, g_L = 0.3, v_{Na} = 115, v_K = -12, v_L = 10.6, I = 20$$

$$a_M = \frac{0.1 \times (25 - V)}{\exp(\frac{25 - V}{10}) - 1}, a_H = 0.07 \times \exp(-\frac{V}{20}), a_N = \frac{0.01 \times (10 - V)}{\exp(\frac{10 - V}{10}) - 1}$$

$$b_M = 4 \times \exp(-\frac{V}{18}), b_H = \frac{1}{\exp(\frac{30 - V}{10}) + 1}, b_N = 0.125 \times \exp(-\frac{V}{80})$$

(6) Cell cycle model by Pomerening *et al.*

state variables: $a, b, c, d, e, f, g, h, i$

time derivatives of state variables: $\frac{da}{dt}, \frac{db}{dt}, \frac{dc}{dt}, \frac{dd}{dt}, \frac{de}{dt}, \frac{df}{dt}, \frac{dg}{dt}, \frac{dh}{dt}, \frac{di}{dt}$

$$\frac{da}{dt} = k_{synth} - k_{dest} \times i \times a - k_a \times (cdk1_{tot} - b - c - d - e) \times a + k_d \times b$$

$$\frac{db}{dt} = k_a \times (cdk1_{tot} - b - c - d - e) \times a - k_d \times b - k_{dest} \times i \times b - k_{wee1} \times g \times b - k_{wee1basal} \times (wee1_{tot} - g) \times b$$
$$+ k_{cdc25} \times f \times c + k_{cdc25basal} \times (cdc25_{tot} - f) \times c$$

$$\frac{dc}{dt} = k_{wee1} \times g \times b + k_{wee1basal} \times (wee1_{tot} - g) \times b - k_{cdc25} \times f \times c - k_{cdc25basal} \times (cdc25_{tot} - f) \times c - k_{cak} \times c + k_{pp2c} \times d - k_{dest} \times i \times$$ [illegible]

$$\frac{dd}{dt} = k_{cak} \times c - k_{pp2c} \times d - k_{cdc25} \times f \times d - k_{cdc25basal} \times (cdc25_{tot} - f) \times d + k_{wee1} \times g \times e + k_{wee1basal} \times (wee1_{tot} - g) \times e - k_{dest} \times i$$ [illegible]

$$\frac{de}{dt} = k_{cdc25} \times f \times d + k_{cdc25basal} \times (cdc25_{tot} - f) \times d - k_{wee1} \times g \times e - k_{wee1basal} \times (wee1_{tot} - g) \times e - k_{dest} \times i \times e$$

$$\frac{df}{dt} = k_{cdc25on} \times \frac{e^{n_{cdc25}}}{ec50_{cdc25}{}^{n_{cdc25}} + e^{n_{cdc25}}} \times (cdc25_{tot} - f) - k_{cdc25off} \times f$$

$$\frac{dg}{dt} = -k_{wee1off} \times \frac{e^{n_{wee1}}}{ec50_{wee1}{}^{n_{wee1}} + e^{n_{wee1}}} \times g + k_{wee1on} \times (wee1_{tot} - g)$$

$$\frac{dh}{dt} = k_{plx1on} \times \frac{e^{n_{plx1}}}{ec50_{plx1}{}^{n_{plx1}} + e^{n_{plx1}}} \times (plx1_{tot} - h) - k_{plx1off} \times h$$

$$\frac{di}{dt} = k_{apcon} \times \frac{h^{n_{apc}}}{ec50_{plx1}{}^{n_{apc}} + h^{n_{apc}}} \times (apc_{tot} - i) - k_{apcoff} \times i$$

$$r = 10, k_{synth} = 0.04, k_{dest} = 0.01, k_a = 0.1, k_d = 0.001, k_{wee1} = 0.05, k_{wee1basal} = \frac{k_{wee1}}{r}$$

$$k_{cdc25} = 0.1, k_{cdc25basal} = \frac{k_{cdc25}}{r}, cdk1_{tot} = 230, cdc25_{tot} = 15, apc_{tot} = 50, plx1_{tot} = 50$$

$$n_{wee1} = 4, n_{cdc25} = 4, n_{apc} = 4, n_{plx1} = 4$$

$$ec50_{plx1} = 40, ec50_{wee1} = 40, ec50_{cdc25} = 40, ec50_{apc} = 40$$

$$k_{cdc25on} = 1.75, k_{cdc25off} = 0.2, k_{apcon} = 1, k_{apcoff} = 0.15, k_{plx1on} = 1, k_{plx1off} = 0.15$$

$$k_{wee1on} = 0.2, k_{wee1off} = 1.75, k_{cak} = 0.8, k_{pp2c} = 0.008, wee1_{tot} = 15$$

(7) cAMP model by Martiel and Goldbeter

State variables: x, y, z

Time derivatives of state variables: $\frac{dx}{dt}, \frac{dy}{dt}, \frac{dz}{dt}$

$$\frac{dx}{dt} = -f1 \times + f2 \times (1 - x)$$

$$\frac{dy}{dt} = q \times \sigma \times \psi - (k_i + k_t) \times y$$

$$\frac{dz}{dt} = \frac{k_t \times y}{h} - k_e \times z$$

$$c = 10, k_1 = 0.036, e = 1, h = 5, a = 3, k_2 = 0.666, L1 = 10, L2 = 0.005$$

$$q = 4000, \sigma = 0.6, k_i = 1.7, k_e = 5.4, k_t = 0.9, \theta = 0.01, \lambda = 0.01$$

$$f1=\frac{k_1+k_2\times z}{1+z},f2=\frac{k_1\times L1+k_2\times L2\times c\times z}{1+c\times z},Y=\frac{x\times z}{1+z}$$
$$\psi=a\times\frac{\lambda\times\theta\times e\times Y^2}{1+a\times\theta+e\times Y^2\times(1+a)}$$

(8) Glycolysis model by Sel'kov

State variables: x, y

Time derivatives of state variables: $\frac{dx}{dt},\frac{dy}{dt}$

$$\frac{dx}{dt}=1-x\times y^{\gamma}$$
$$\frac{dy}{dt}=\alpha\times y\times(x\times y^{\gamma-1}-1)$$

$\gamma=2,\alpha=1.1$

(9) Glycolysis model by Higgins (modified version)

State variables: x, y, z

Time derivatives of state variables: $\frac{dx}{dt},\frac{dy}{dt},\frac{dz}{dt}$

$$\frac{dx}{dt}=v1-\frac{x\times y}{1+y\times(1+x)}$$
$$\frac{dy}{dt}=\alpha\times\frac{x\times y}{1+y\times(1+x)}-v2\times\frac{y}{k+y}$$

$v1=0.1,v2=0.15,k=0.3,\alpha=10$

IV. Additional Notes

Additional Note A1. Advantages of interaction perturbation over parameter perturbation in this study

The purpose of this study is to investigate the different regulatory functions of networks arising from the differences in their structure. This purpose can be achieved by examining how a network responds to a perturbation of each link within the network. Then, a question is raised as to whether a parameter perturbation in an ODE model can be regarded as a link perturbation.

The number and the function of the parameters are determined by the structure of the ODE model. For instance, in a network structure, 'Y inhibit X' can be represented by the equation (1.1) as well as by the equation (1.2). Y inhibits the synthesis of X in the equation (1.1) while Y facilitates the degradation of X in the equation (1.2).

$$\frac{dX}{dt} = \frac{k_{sx}}{K_m + Y} - k_{dx} \times X \quad (1.1)$$

$$\frac{dX}{dt} = k_{sx} - k_{dx} \times Y \times X \ (1.2)$$

There are three parameters (i.e. k_{sx}, K_m, and k_{dx}) in the equation (1.1) and two parameters (i.e. k_{sx} and k_{dx}) in the equation (1.2) although those two equations represent the same network structure which consists of two links (i.e. 'X on X' and 'Y on X'). In the equation (1.1), k_{dx} represents 'X on X'; k_{sx} and K_m represent 'Y on X'. Herein, the perturbation of k_{dx} clearly means the perturbation of the link 'X on X'.

However, in the link 'Y on X', the perturbation of either k_{sx} or K_m may not exactly correspond to the perturbation of the link. In the equation (1.2), k_{dx} is involved in two links (i.e. 'X on X' and 'Y on X') simultaneously. In this structure, it is impossible to perturb a link independently of other links by parameter perturbation.

In summary, more than two parameters may represent a single link and a single parameter may be

involved in more than two links. Therefore, this problem may undermine the reliability of the results of parameter perturbations.

Direct modulation of specific interaction can be a solution to this problem. All the ODE models which represent 'Y inhibits X' will consist of two interactions (i.e. X on X and Y on X). Herein 'Y inhibits X' represents the negative interaction from Y to X. What will be the results when we directly modulate the interaction from Y to X? In the equation (1.1), $\frac{dX}{dt}$ will increase due to the increased first term ($\frac{k_{sx}}{K_m + Y}$). In the equation (1.2), $\frac{dX}{dt}$ will also increase due to the decreased second term ($k_{dx} \times Y \times X$). Regardless of the structure of ODE models, the results of the direct perturbation of an interaction reflect the link structure inside the network. Therefore, perturbation of interactions will be methodologically suitable for analyses of the different functions of networks arising from their structure. In this study, interactions between molecules were represented algebraically by using Jacobian matrix and the functional characteristics of the networks were investigated by direct perturbation of interactions.

Additional Note A2. Difference between the first-order ODE and the second-order ODE in respect of responses to a perturbation of the system

In the first-order ODE, applying transient perturbation does not change the frequency and amplitude of the limit cycle oscillator. However, the transient perturbation in the second-order ODE can change them. This is because, for the second-order ODE, the first derivatives of the state variables as well as the state variables themselves change their values during the integration process.

Suppose that the system is defined by coupled ODEs with two state variables, X and Y, for convenience of explanation. If it is a first-order ODE system, we need two initial conditions (i.e., X(t=0) and Y(t=0)) and parameter values for numerical simulation. In this case, the simulation using

identical parameter values and appropriate initial conditions would generally lead the system to converge to a same fixed point (or limit cycle). In contrast, for the simulation of an equivalent second-order ODE system, the number of required initial conditions becomes double compared to that for the first-order ODE system (i.e., X(t=0), Y(t=0), $\frac{dX}{dt}(t=0), \frac{dY}{dt}(t=0)$). In this case, the simulation using identical parameter values might not converge to a same fixed point (or limit cycle) even if the initial conditions for X and Y (i.e., X(t=0), Y(t=0)) are identical. This is because the additionally required initial conditions (i.e., $\frac{dX}{dt}(t=0), \frac{dY}{dt}(t=0)$) are different. In Figure R1 shown below, we presented the simulation results that show a difference between a first-order ODE system and the equivalent second-order ODE system: different limit cycles are generated by integrations of the equivalent second-order ODE system with the same parameter set and same initial values of the state variables, but with different initial time derivatives of the state variables.

These results indicate that every time when the interaction perturbation is performed, the second-order ODE system may reach a different initial condition (i.e., different initial values and different time derivatives), consequently leading to a different limit cycle.

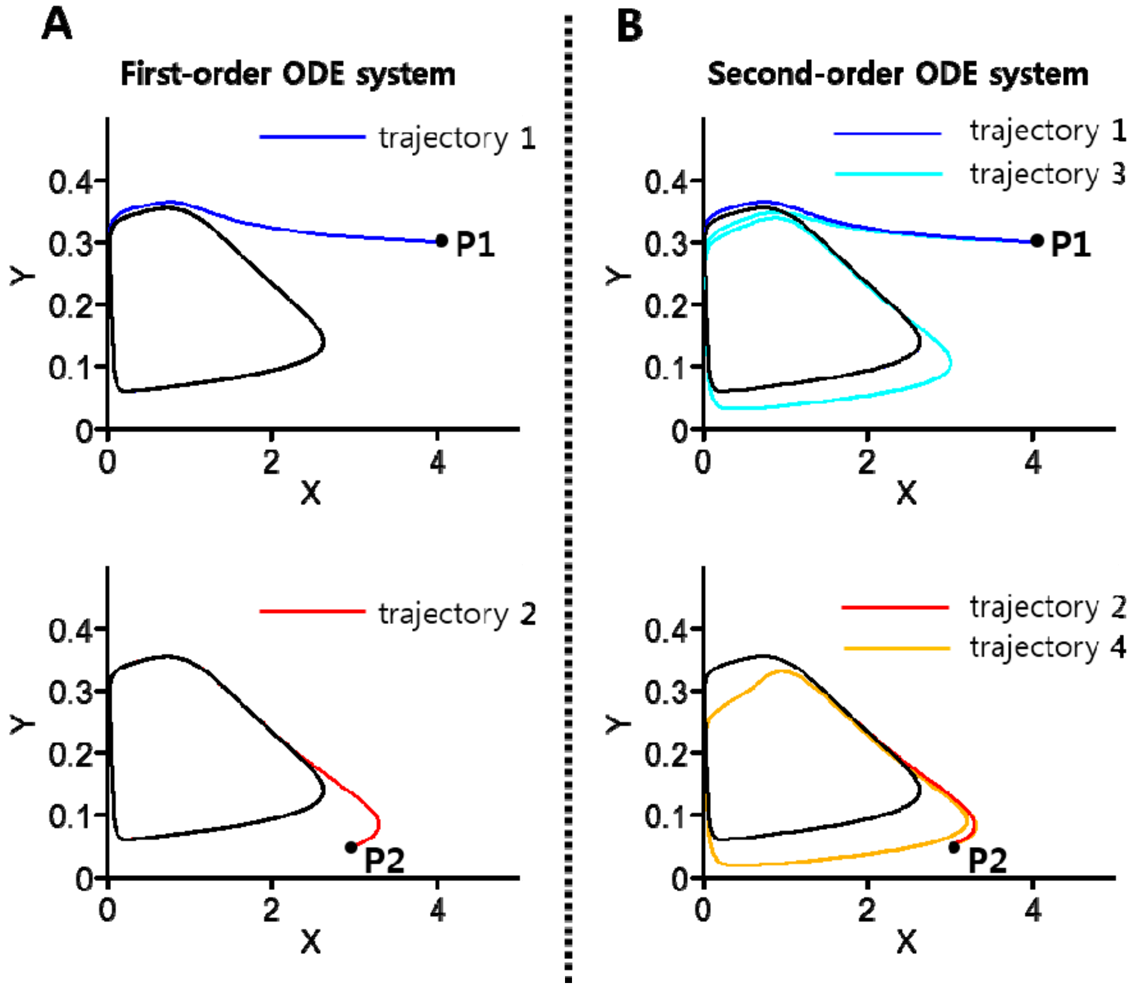

Figure R1. Comparison between a first-order ODE system and the equivalent second-order ODE system of a two-node activator amplified NFO. (A) Simulation results of the first-order ODE system. Integrations from two different initial values (i.e., P1, P2) lead to the same limit cycle. (B) Simulation results of the equivalent second-order ODE system. Integrations from the same initial values (e.g. P1) can result in different limit cycles due to the different initial time derivatives of the state variables (e.g. trajectory 1 and trajectory 3: their limit cycles are different). Detailed information on the simulation is provided in Table R1.

Table R1. Equations, parameters, and initial conditions for the simulation of a two-node activator amplified NFO.

	First-order ODE system	Equivalent second-order ODE system
Equations	$\frac{dx}{dt} = k_{sx\_prime} + k_{sx} \times \frac{x^q}{1+x^q} - (k_{dx\_prime} + k_{dx} \times y) \times x$ $\frac{dy}{dt} = k_{sy} \times \frac{x^p}{1+x^p} - k_{dy} \times y$	$\frac{d^2x}{dt^2} = k_{dx}x(k_{dy}y - \frac{k_{sy}x^p}{x^p+1})$ $-(k_{sx\_prime} - x(k_{dx\_prime} + k_{dx}y) + \frac{k_{sx}x^q}{x^q+1}) \times s$ $(k_{dx\_prime} + k_{dx}y - \frac{k_{sx}qx^{q-1}}{x^q+1} + \frac{k_{sx}qx^{2q-1}}{(x^q+1)^2})$ $\frac{d^2y}{dt^2} = k_{dy}(k_{dy}y - \frac{k_{sy}x^p}{x^p+1}) + (\frac{k_{sy}px^{p-1}}{x^p+1} - \frac{k_{sy}px^{2p-1}}{(x^p+1)^2}) \times$ $(k_{sx\_prime} - x(k_{dx\_prime} + k_{dx}y) + \frac{k_{sx}x^q}{x^q+1})$
Parameters	$k_{sx\_prime} = 0.02,\ k_{sx} = 1,\ k_{dx\_prime} = 0.2,\ k_{dx} = 1,$ $q = 2,\ k_{sy} = 0.01,\ k_{dy} = 0.01,\ p = 2$	$k_{sx\_prime} = 0.02,\ k_{sx} = 1,\ k_{dx\_prime} = 0.2,\ k_{dx} = 1,$ $q = 2,\ k_{sy} = 0.01,\ k_{dy} = 0.01,\ p = 2$
Initial conditions	For trajectory 1, $(x_{t=0}, y_{t=0}) = (4, 0.3)$ For trajectory 2, $(x_{t=0}, y_{t=0}) = (3, 0.05)$	For trajectory 1, $(x_{t=0}, y_{t=0}) = (4, 0.3)$ $(\frac{dx}{dt}_{t=0}, \frac{dy}{dt}_{t=0}) = (-1.0388, 0.0064)$ For trajectory 2, $(x_{t=0}, y_{t=0}) = (3, 0.05)$ $(\frac{dx}{dt}_{t=0}, \frac{dy}{dt}_{t=0}) = (0.1700, 0.0085)$ For trajectory 3, $(x_{t=0}, y_{t=0}) = (4, 0.3)$ $(\frac{dx}{dt}_{t=0}, \frac{dy}{dt}_{t=0}) = (-1.0378, 0.0054)$

		For trajectory 4,<br><br>$(x_{t=0}, y_{t=0}) = (3, 0.05)$<br><br>$(\frac{dx}{dt}_{t=0}, \frac{dy}{dt}_{t=0}) = (0.1685, 0.0070)$
